# Tumor-associated macrophage, angiogenesis and lymphangiogenesis markers predict prognosis of non-small cell lung cancer patients

**DOI:** 10.1186/s12967-020-02618-z

**Published:** 2020-11-23

**Authors:** Ilseon Hwang, Jeong Won Kim, Kris Ylaya, Eun Joo Chung, Haruhisa Kitano, Candice Perry, Jun Hanaoka, Junya Fukuoka, Joon-Yong Chung, Stephen M. Hewitt

**Affiliations:** 1 Experimental Pathology Laboratory, Laboratory of Pathology, Center for Cancer Research, National Cancer Institute, National Institutes of Health, MSC1500, Bethesda, MD 20892 USA; 2 Radiation Oncology Branch, Center for Cancer Research, National Cancer Institute, National Institutes of Health, Bethesda, MD 20892 USA; 3 Department of Thoracic Surgery, Vories Memorial Hospital, Shiga, 523-0806 Japan; 4 Advanced Biomedical Computational Science, Biomedical Informatics and Data Science, Leidos Biomedical Research, Inc., Frederick, MD 21702 USA; 5 Department of Thoracic Surgery, Shiga University of Medical Science, Otsu, 520-2192 Japan; 6 Department of Pathology, Nagasaki University Graduate School of Biomedical Sciences, Nagasaki, 852-8523 Japan; 7 Department of Pathology, Keimyung University Scholl of Medicine and Institute for Cancer Research, Dongsan Medical Center, Daegu, 42601 Republic of Korea; 8 Department of Pathology, Kangnam Sacred Heart Hospital, Hallym University College of Medicine, Seoul, 07441 Republic of Korea

**Keywords:** Tumor-associated macrophage, CD163+/CD68+ ratio, Vascular endothelial growth factor, Angiogenesis, Lymphangiogenesis, Non-small cell lung cancer, Prognosis

## Abstract

**Background:**

The tumor microenvironment (TME) is a critical player in tumor progression, metastasis and therapy outcomes. Tumor-associated macrophages (TAMs) are a well-recognized core element of the TME and generally characterized as M2-like macrophages. TAMs are believed to contribute to tumor progression, but the mechanism behind this remains unclear. We aimed to investigate the clinical, angiogenic, and lymphangiogenic significance of TAMs in non-small cell lung cancer (NSCLC).

**Methods:**

Utilizing combined immunohistochemistry and digital image analysis, we assessed CD68, CD163, VEGF-A, and VEGF-C expression in 349 patients with NSCLC. Subsequently, the potential association between M2 TAMs and angiogenic VEGF-A and/or lymphangiogenic VEGF-C was evaluated for its prognostic value. Furthermore, the effects of M2 TAMs on angiogenesis and lymphangiogenesis were explored via an in vitro co-culture system.

**Results:**

CD68 and CD163 expression were found to directly correlate with VEGF-A and/or VEGF-C expression (all *p* < 0.001). Furthermore, elevated M2 ratio (CD163+/CD68+) was significantly associated with poor overall survival (*p* = 0.023). Dual expression of M2 ratio^high^ and VEGF-C^high^ (M2 ratio^high^VEGF-C^high^) was correlated with worse overall survival (*p* = 0.033). Multivariate analysis revealed that M2 ratio^high^ [HR (95% CI) = 1.53 (1.01–2.33), *p* = 0.046] and combined M2 ratio^high^VEGF-C^high^ expression [HR (95% CI) = 2.01 (1.28–3.16), *p* = 0.003] were independent predictors of poor overall survival. Notably, we confirmed that M2 macrophages significantly enhanced the protein and mRNA expression of both VEGF-A and VEGF-C, while M1 macrophages induced only mRNA expression of *VEGF-A* in A549 cells.

**Conclusions:**

This study suggests that TAMs are significantly associated with angiogenesis and lymphangiogenesis, contributing to the progression of NSCLC. Furthermore, elevated M2 ratio, similar to combined high M2 ratio and high VEGF-C expression, is a strong indicator of poor prognosis in patients with NSCLC, providing insight for future TAM-based immunotherapy strategies.

## Background

Lung cancer is the primary cause of cancer-related deaths worldwide, and is characterized by a poor prognosis in its advanced stages [1]. Non-small cell lung cancer (NSCLC) accounts for approximately 85% of reported lung cancer cases with most new NSCLC cases diagnosed at advanced stages [2]. The tumor, node, metastasis (TNM) staging system approved by the International Association for the Study of Lung Cancer (IASLC) and the American Joint Committee on Cancer (AJCC) is used internationally to characterize the extent of disease and its correlations with survival. Thus, current NSCLC treatments are largely guided by TNM stages. Despite the availability of various combined treatments, the overall survival rate of NSCLC patients remains poor, with only 68% of patients with stage IB and under 10% of patients with stage IVA-B surviving 5 years post diagnosis. Most deaths during stage III of NSCLC are caused by metastatic recurrence after surgical resection. Furthermore, an estimated 80% of patients with NSCLC receive an initial diagnosis after their cancer has already spread to regional lymph nodes or metastasized to distant organs [3]. Recently, immune checkpoint inhibitors have been an important component in the management of advanced NSCLC because they have led to improved survival and antitumor response in comparison to that of conventional chemotherapy. Unfortunately, a very limited number of select patients with advanced NSCLC benefitted from such treatment. Currently, programmed death ligand 1 (PD-L1) expression on tumor cells [4, 5], tumor mutation burden (TMB) [6, 7], tumor-infiltrating lymphocytes (TILs) [8], microsatellite instability (MSI) [9], tumor microenvironment (TME) [10], and microbiome [11] are factors taken under consideration when administering immune checkpoints inhibitors to NSCLC patients. Still, less than 30% of patients respond to this treatment. Thus, more effective biomarkers are necessary to better predict the effectiveness of immunotherapy and improve risk stratification before treatment.

It is known that leukocytes, including macrophages, infiltrate tumor tissues and form the TME with fibroblasts and vascular endothelial cells. Tumor-associated macrophages (TAMs) are highly plastic and can alter their phenotype (M1 pro-inflammatory or M2 anti-inflammatory) according to location and surrounding cytokine milieu in the TME. M1 macrophages are considered to be key players in recognition and destruction of cancer cells [12] whereas M2 macrophages are thought to help promote tumor growth and metastasis in the periphery of solid tumors [13]. CD68 is a pan-macrophage marker, whereas CD163 is a specific marker for the M2 subpopulation [13]. Prior studies have shown that a high density of TAMs is linked to poor patient prognosis in many cancers [14], but other studies have reported contrary results [15, 16]. Thus, the association between TAMs and cancer prognosis remains controversial.

Angiogenesis and lymphangiogenesis play a critical role in tumor growth and metastasis in NSCLC [17]. Angiogenesis refers to the development and growth of new blood vessels, which support tumor growth, as well as tumor invasion and metastasis, by providing oxygen, nutrients and growth factors. Previous studies have demonstrated that TAMs promote proangiogenic factors in malignant tumors, creating a suitable microenvironment for angiogenesis [18–20]. On the other hand, lymphangiogenesis, the process of forming new lymphatic vessels, is the key initial step in lymphatic and regional lymph node metastasis [21]. The significant association between TAM density and tumor lymphatic vessel density was confirmed in several cancers, including lung cancer [22]. Among the vascular endothelial growth factor (VEGF) family members, VEGF-A and VEGF-C are considered to be major mediators of tumor angiogenesis and lymphangiogenesis, respectively [23]. TAMs are an important driver of angiogenesis and lymphangiogenesis; however, the mechanism of this process remains unclear. Therefore, in this study, we aimed to evaluate stroma-infiltrating macrophages (M1 and M2 macrophages), VEGF-A, and VEGF-C expression by immunohistochemistry (IHC) and quantitative digital image analysis. Furthermore, we analyzed the potential association between M2 TAMs and angiogenesis and/or lymphangiogenesis in patients with NSCLC.

## Methods

### Tissue samples

A total of 349 surgically resected primary NSCLC specimens were collected. This includes samples from South Korean patients (*n* = 102) who underwent curative surgery and adjuvant chemotherapy at Keimyung University Dongsan Medical Center between January 2010 and December 2012, and samples (*n* = 247) from Japanese patients who underwent curative resections between 1993 and 2004 at Toyama University Hospital and National Hospital Organization Higashi-Ohmi General Medical Center, as previously reported [24]. TNM classification of NSCLC tumors were staged according to the eighth edition Lung Cancer standards [25], Grading was done according to the 2015 World Health Organization (WHO) guidelines. Clinicopathological characteristics and clinical outcome data were retrospectively collected from medical records and pathology reports. The median follow-up period for the Korean and Japanese cohorts was 42.6 months (range 0.7—68.5 months) and 28.0 months (range 0–311.0 months), respectively. This study was approved by the Institutional Review Board at Keimyung University Dongsan Medical Center (DSMC 2020-01-020, Daegu, Republic of Korea), Toyama University Hospital (Toyama, Japan), and National Hospital Organization Higashi-Ohmi General Medical Center (Shiga, Japan).

### Tissue microarray and immunohistochemistry

Tissue Microarray (TMA) was constructed from archival formalin-fixed, paraffin-embedded (FFPE) tissue blocks. Three 1.0 mm diameter tissue cores were arrayed on a recipient paraffin block using a tissue arrayer (Pathology Devices, Westminster, MD), in which a representative tumor area was carefully selected for each tumor from a hematoxylin and eosin (H&E) stained section of a donor block. TMA blocks were cut into serial 5-µm-thick sections, heated for 1 h at 60 ℃, deparaffinized in xylene, and rehydrated through a series of graded alcohol to distilled water. Heat mediated antigen retrieval was performed in a pressure chamber (Pascal; Dako, Carpinteria, CA) with pH 6.0 citrate buffer (Dako) for CD68, CD163 and VEGF-C, but pH 9.0 citrate buffer (Dako) for VEGF-A. Endogenous peroxidase activity was quenched using a 3% solution of aqueous hydrogen peroxide and non-specific binding was blocked with an additional protein block (Dako). Subsequently, primary antibody hybridization was carried out with the following: mouse monoclonal anti-CD68 (Clone Kp1; diluted 1:5000; Dako) for 30 min, rabbit monoclonal anti-CD163 (clone EPR19518, diluted 1:1000; Abcam, Cambridge, MA) for 1 h, mouse monoclonal anti-VEGF-A (clone VG1; diluted 1:100; Thermo Fisher Scientific, Waltham, MA) for 1 h; goat polyclonal anti-VEGF-C (Cat.# AF752; diluted 1:100; R&D Systems, Minneapolis) at 4 ℃ overnight incubation. Signals were detected with an Envision + detect system (Dako). The stains were visualized using 3,3′-diaminobenzidine (DAB), lightly counterstained with hematoxylin, dehydrated in ethanol, and cleared in xylene. In a similar manner, dual staining of CD68 and CD163 was performed using a CINtec Plus Cytology Kit (CINtec PLUS; Roche, Indianapolis, IN), modified for FFPE tissue-based specimens. Briefly, endogenous activity was blocked with hydrogen peroxidase, followed by an incubation with a primary antibody cocktail, consisting of mouse anti-CD68 and rabbit anti-CD163, for 1 h at an aforementioned dilution. Multicolor brown/red enzymatic reactions were detected using a horseradish peroxidase (HRP) and alkaline phosphatase (AP) polymer-based system (CINtec PLUS), then proceeded with DAB and substrate Red chromogen labeling. After rinsing and light hematoxylin counter staining, slides were allowed to air dry, briefly cleared in xylene, and coverslipped. Immunoglobulin G (IgG) isotype and omission of the primary antibodies were used as negative controls. Positive controls were in TMA including testis tissues.

### Digital image analysis

Immunohistochemically stained slides were scanned using an Aperio AT2 digital scanner with a 40× objective (Leica Biosystems Inc., Buffalo Grove, IL). The images were analyzed using Visiopharm Digital Image Analysis (DIA) software (for Windows 7, version 6.9.1; Visiopharm, Hørsholm, Denmark). The cytoplasm was defined by outlining the nucleus with a system trained to digitally “paint” cell nuclei. The proportion of positively brown-stained cells was obtained using a predefined algorithm and optimized settings, as previously described [26]. The immunohistochemical score was expressed as the percentage of positive cells (possible range 0–100). The median values were used as cut-off values for discriminating between low and high expression of immunohistochemical staining. Cut-off values for CD68, CD163, VEGF-A, VEGF-C, and M2 ratio (CD163+/CD68+) were 8.70%, 10.12%, 7.42%, 3.09%, and 1.17 with high cytoplasmic staining, respectively.

### Cell culture

THP-1 and A549 cells were purchased from the ATCC (Manassas, VA) and cultured in RPMI 1640 (Invitrogen, Carlsberg, CA), containing 10% of heat inactivated fetal bovine serum (Invitrogen), in a 37 °C CO_2_ incubator. The culture medium for THP-1 cells was supplemented with 0.05 mM *ß*-mercaptoethanol (Gibco, 31350–010; Thermo Fisher Scientific). For differentiation of monocytic THP-1 cells towards macrophage (M0) phenotype, cells were incubated with 150 nM phorbol 12-myristate 13-acetate (PMA, P8139; Millipore Sigma, Burlington, MA) for 24 h. Differentiated macrophages were polarized toward M1 or M2 macrophages.

### Co-culture with A549 cells and polarized macrophages

For co-culture experiments with A549 cells and polarized macrophages, differentiated M0 macrophages from THP-1 were transferred into a 12-transwell insert (2 × 10^5^ cells /insert, membrane pore size of 0.4 μm, Corning, #3450) and treated with 10 pg/ml of lipopolysaccharide (LPS; Sigma, #8630), or 20 ng/ml of interleukin 4 (R&D Systems, #204-IL) and 20 ng/ml of interleukin 13 (R&D Systems, #213-ILB), for 72 h. A549 cells were seeded into a new 12-well plate (2 × 10^4^ cells/well) and incubated in RPMI containing 10% FBS, 24 h prior to co-culture. Polarized macrophages were washed with PBS three times in transwell inserts and co-cultured with A549 cells already plated in a new 12-well plate. After 48 h, only cells were collected for further experiments such as quantitative real-time polymerase chain reaction (PCR) or enzyme-linked immunosorbent assay (ELISA).

### ELISA

Proteins were prepared from collected cells, and VEGF-A and VEGF-C protein levels were quantified in cell lysates (100 µg of total protein) by using specific ELISA kits (R&D system, Minneapolis, MN) according to the manufacturer’s instruction.

### Quantitative real-time PCR

To assess *VEGF-A* and *VEGF-C* mRNA levels, total RNA was extracted from macrophages, derived from THP-1 cells, and A549 cells using a Qiagen RNeasy Mini kit (Qiagen, Valencia, CA), then converted to cDNA using a QuantiTech Reverse Transcriptase kit (Qiagen) according to the manufacturer’s protocols. Quantitative real-time PCR was performed with 0.5 µg of cDNA assayed in a 50 μL reaction volume. The reactions were incubated for 2 min at 50 °C, 10 min at 95 °C for initial denaturing, then run through 40 cycles of 95 °C for 15 s and 60 °C for 1 min in 7500 Taqman assays from ABI (Applied Biosystems, Foster City, CA).

### Statistical analysis

Statistical analyses were performed using R 3.5.2 (R Development Core Team, Vienna, Austria, https://www.R-project.org) and the SPSS Statistics for Windows, version 23 (IBM Corp., Armonk, NY). Differences in clinicopathological features between low and high expression of CD68, CD163, VEGF-A and VEGF-C were analyzed using Chi-square or Fisher’s exact test for categorical variables and the Student’s t test for continuous variables. Survival rate was determined by the Kaplan–Meier method, and the log-rank test was used to compare survival rates among subgroups. The log-rank test was used for univariate analysis and independent prognostic factors were identified by multivariate analysis, using the Cox proportional hazards model to calculate hazard ratios.

At first, pathologic T stage, N stage and M2 ratio (CD163+/CD68+) were included as covariates, and then pathologic T stage, N stage and M2 ratio/VEGF-C were included as covariates. The results of the Cox model analysis were reported using hazard ratios and 95% confidence intervals (CIs). *P* values of less than 0.05 were defined as indicators of statistically significant differences.

## Results

### Clinicopathological characteristics of patients

The study group was composed of 241 (69.1%) males and 108 (30.9%) females with a mean age of 65.5 ± 8.59 years (range 35 to 90 years). There were 210 (60.2%) patients with adenocarcinoma, 135 (38.7%) patients with squamous cell carcinoma, and 4 (1.1%) patients with adenosquamous carcinoma. Histologically, 125 (35.8%) tumors were graded as well-differentiated, 158 (45.3%) as moderately differentiated, and 66 (18.9%) as poorly differentiated. Stage pT1, pT2, pT3, and pT4 tumors were identified in 155 (44.4%), 148 (42.4%), 43 (12.3%), and 3 (0.9%) patients, respectively. Lymph node metastasis occurred in 102 patients (29.2%); pN stage was pN1 in 52.9% (54/102), pN2 in 45.1% (46/102), and pN3 in 2.0% (2/102) of patients. Stage I, II, III, and IV lesions at initial diagnosis were present in 218 (62.5%), 72 (20.6%), 52 (14.9%), and 7 (2.0%) patients, respectively. Of the 349 patients, 93 (26.6%) had died at the time of analysis. The overall 5-year survival rate was 45.9%. The clinicopathological characteristics of the patients are summarized in Additional file 1: Table S1.

### Expression of TAM, angiogenesis and lymphangiogenesis markers

**Fig. 1:**
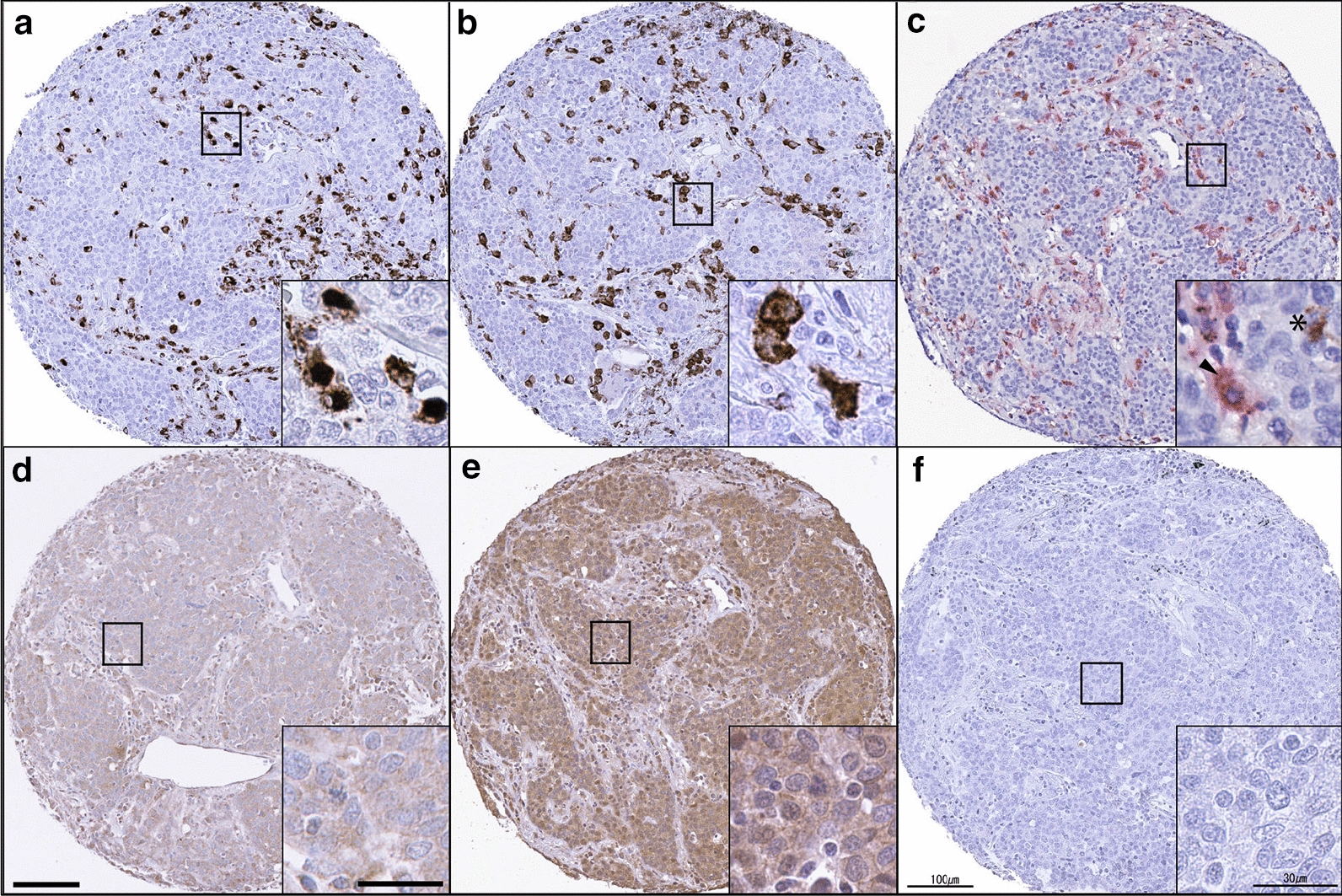
Tumor-associated macrophage markers (CD68 & CD163), VEGF-A, and VEGF-C expression in human non-small cell lung cancer (NSCLC) tissues. Representative immunohistochemical images of CD68 (**a**), CD163 (**b**), double staining of CD68 and CD163 (**c**), VEGF-A (**d**), and VEGF-C (**e**), immunoglobulin G (IgG) isotype control (**f**). Arrowhead indicates dual stained CD163 (red) and CD68 (brown), and asterisk indicates CD68. Scale bar = 100 µm (30 µm in inset)

CD68 and CD163 positive macrophages were predominantly detected in the tumor stroma and intervening space between tumor cells. We confirmed the location of CD68 and CD163 positive macrophages using CD68 and CD163 dual staining (Fig. 1a–c). Immunohistochemical staining of VEGF-A and VEGF-C identified their presence in tumor cells and mesenchymal cells in the stroma (Fig. 1d, e). A total of 174 (49.9%) tumors were classified as samples highly expressing CD68 (CD68^High^), CD163 (CD163^High^), and VEGF-A (VEGF-A^High^), while 175 (50.1%) tumors were determined to highly express VEGF-C (VEGF-C^High^) (Table 1).

**Table 1:** Clinicopathological parameters of patients with CD68, CD163, VEGF-A, or VEGF-C expression in patients with NSCLC

Category (*n* = 349)	CD68			CD163			CD163 + /CD68 + (M2 ratio)			VEGF-A			VEGF-C		
	High *n* (%)	Low *n* (%)	*p*	High *n* (%)	Low *n* (%)	*p*	High *n* (%)	Low *n* (%)	*p*	High *n* (%)	Low *n* (%)	*p*	High *n* (%)	Low *n* (%)	*p*
Total	174 (49.9)	175 (50.1)		174 (49.9)	175 (50.1)		174 (49.9)	175 (50.1)		174 (49.9)	175 (50.1)		175 (50.1)	174 (49.9)	
Age			0.816			0.183			0.816			0.418			0.583
< 60	36 (20.7)	39 (22.3)		43 (24.7)	32 (18.3)		36 (20.7)	39 (22.3)		41 (23.6)	34 (19.4)		35 (20.0)	40 (23.0)	
≥ 60	138 (79.3)	136 (77.7)		131 (75.3)	143 (81.7)		138 (79.3)	136 (77.7)		133 (76.4)	141 (80.6)		140 (80.0)	134 (77.0)	
Sex			1.000			0.587			0.017			0.314			0.001
Male	120 (69.0)	121 (69.1)		123 (70.7)	118 (67.4)		131 (75.3)	110 (62.9)		125 (71.8)	116 (66.3)		136 (77.7)	105 (60.3)	
Female	54 (31.0)	54 (30.9)		51 (29.3)	57 (32.6)		43 (24.7)	65 (37.1)		49 (28.2)	59 (33.7)		39 (22.3)	69 (39.7)	
Tumor type			0.231			0.027			0.585			0.015			< 0.001
SqCC	74 (42.5)	61 (34.9)		79 (45.4)	56 (32.0)		72 (41.4)	63 (36.0)		80 (46.0)	55 (31.4)		89 (50.9)	46 (26.4)	
AD	99 (56.9)	111 (63.4)		94 (54.0)	116 (66.3)		100 (57.5)	110 (62.9)		93 (53.4)	117 (66.9)		86 (49.1)	124 (71.3)	
ADSq	1(0.6)	3 (1.7)		1 (0.6)	3 (1.7)		2 (1.1)	2 (1.1)		1 (0.6)	3 (1.7)		0	4 (2.3)	
Grade			0.030			0.360			0.446			0.446			0.001
Well	51 (29.3)	74 (42.3)		56 (32.2)	69 (39.4)		68 (39.1)	57 (32.6)		58 (33.3)	67 (38.3)		47 (26.9)	78 (44.8)	
Moderate	84 (48.3)	74 (42.3)		84 (48.3)	74 (42.3)		75 (43.1)	83 (47.4)		79 (45.4)	79 (45.1)		95 (54.2)	63 (36.2)	
Poor	39 (22.4)	27 (15.4)		34 (19.5)	32 (18.3)		31 (17.8)	35 (20.0)		37 (21.3)	29 (16.6)		33 (18.9)	33 (19.0)	
pT stage			0.417			1.000			1.000			0.891			0.891
T1-2	148 (85.1)	155 (88.6)		151 (86.8)	152 (86.9)		151 (86.8)	152 (86.9)		152 (87.4)	151 (86.3)		151 (86.3)	152 (87.4)	
T3-4	26 (14.9)	20 (11.4)		23 (13.2)	23 (13.1)		23 (13.2)	23 (13.1)		22 (12.6)	24 (13.7)		24 (13.7)	22 (12.6)	
pN stage			0.580			0.750			0.533			0.580			0.208
0	126 (72.4)	121 (69.1)		125 (71.8)	122 (69.7)		120 (69.0)	127 (72.6)		126 (72.4)	121 (69.1)		118 (67.4)	129 (74.1)	
1–3	48 (27.6)	54 (30.9)		49 (28.2)	53 (30.3)		54 (31.0)	48 (27.4)		48 (27.6)	54 (30.9)		57 (32.6)	45 (25.9)	
Stage			0.659			0.914			0.125			0.407			0.106
I	110 (63.2)	108 (61.7)		111 (63.8)	107 (61.1)		108 (62.1)	110 (62.9)		112 (64.4)	106 (60.6)		100 (57.1)	118 (67.8)	
II	34 (19.5)	38 (21.7)		36 (20.7)	36 (20.6)		34 (19.5)	38 (21.7)		38 (21.8)	34 (19.4)		45 (25.7)	27 (15.6)	
III	25 (14.4)	27 (15.4)		24 (13.8)	28 (16.0)		31 (17.8)	21 (12.0)		22 (12.6)	30 (17.1)		26 (14.9)	26 (14.9)	
IV	5 (2.9)	2 (1.2)		3 (1.7)	4 (2.3)		1 (0.6)	6 (3.4)		2 (1.2)	5 (2.9)		4 (2.3)	3 (1.7)	

*NSCLC* non-small cell lung cancer, *AD* adenocarcinoma, *SqCC* squamous cell carcinoma, *ADSq* adenosquamous carcinoma, *VEGF* vascular endothelial growth factor

Table 1 depicts the correlations among CD68, CD163, M2 ratio (CD163+/CD68+), VEGF-A, VEGF-C, and clinicopathologic features. Male patients frequently had a higher M2 ratio than female patients (*p* = 0.017). CD163^High^, VEGF-A^High^ and VEGF-C^High^ expression were significantly associated with the histological type of squamous cell carcinoma (*p* = 0.027, *p* = 0.015, and *p* < 0.001, respectively). CD68^High^ and VEGF-C^High^ correlated with less differentiated tumors (*p* = 0.030 and *p* = 0.001, respectively). There was no association between CD68, CD163, VEGF-A, and VEGF-C expressions and NSCLC patient’s age, pathological T and N status, and stage.

Subgroup analysis according to M2 ratio and VEGF-A or VEGF-C expression status (Additional file 1: Tables S2 and S3) revealed that combined M2 ratio^High^VEGF-A^High^ and combined M2 ratio^High^VEGF-C^High^ were predominantly found in males (*p* = 0.022 and *p* < 0.001, respectively). Combined M2 ratio^High^VEGF-C^High^ was linked with squamous cell carcinoma (*p* < 0.001), while combined M2 ratio^Low^VEGF-C^Low^ was linked with well differentiated tumors (*p* = 0.010). In the squamous cell carcinoma subgroup, VEGF-C^High^ was associated with less differentiation (*p* = 0.029) (Additional file 1: Table S4). However, no significant association was observed in the adenocarcinoma group (Additional file 1: Table S5).

### Correlation between TAMs and VEGF-A or VEGF-C expression

**Fig. 2:**
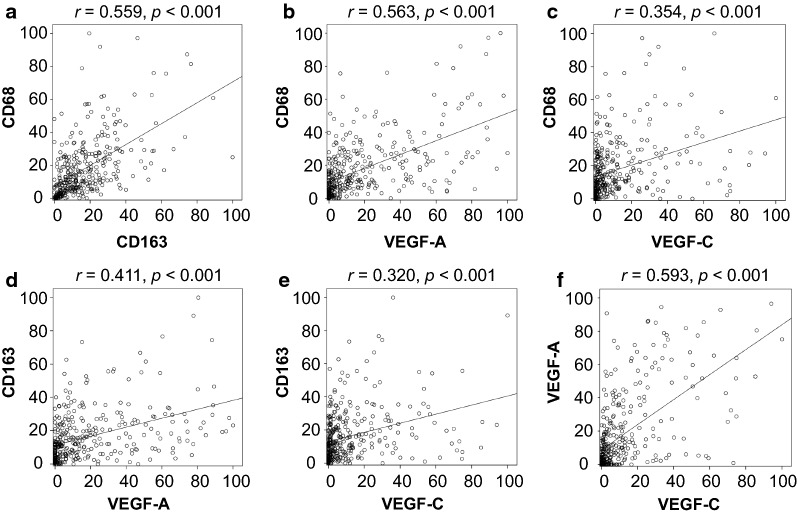
Correlation among tumor-associated macrophage-, angiogenesis- and lymphangiogenesis-related markers in patients with NSCLC. CD68 expression positively correlated with CD163 (**a**), VEGF-A (**b**), and VEGF-C (**c**) expression. CD163 expression showed a significant positive correlation with VEGF-A (**d**) and VEGF-C (**e**) expression. There is a strong positive correlation between VEGF-A and VEGF-C (**f**)

CD68 expression showed a significant positive correlation with CD163 (*Pearson correlation r* = 0.559, *p* < 0.001, Fig. 2a) and VEGF-A expression (*Pearson correlation r* = 0.563, *p* < 0.001), while a moderate correlation was observed between CD68 and VEGF-C expression (*Pearson correlation r* = 0.354, *p* < 0.001, Fig. 2c). CD163 expression moderately correlated with VEGF-A (*Pearson correlation r* = 0.411, *p* < 0.001, Fig. 2d) and VEGF-C (*Pearson correlation r* = 0.320, *p* < 0.001, Fig. 2e) as well. There was a strong correlation between VEGF-A and VEGF-C expression (*Pearson correlation r* = 0.593, *p* < 0.001, Fig. 2f). Furthermore, VEGF-A and VEGF-C protein expression directly correlated with elevated CD68 and CD163 expression (Additional file 2: Fig. S1).

In subgroup analyses, CD68 expression was strongly correlated with CD163 (*Pearson correlation r* = 0.636, *p* < 0.001) and VEGF-A (*Pearson correlation r* = 0.533, *p* < 0.001) expression, while there was a moderate correlation between CD68 and VEGF-C (*Pearson correlation r* = 0.329, *p* < 0.001) expression (Additional file 2: Fig. S2) in adenocarcinoma patients. CD163 expression also significantly correlated with VEGF-A expression (*Pearson correlation r* = 0.459, *p* < 0.001). On the other hand, there was a weak correlation between CD163 and VEGF-C (*Pearson correlation r* = 0.275, *p* < 0.001) expression (Additional file 2: Fig. S2) in adenocarcinoma cases. Similar correlation coefficient values were observed in squamous cell carcinoma patients (Additional file 2: Fig. S3). There were significant correlations between CD68 and CD163 (*Pearson correlation r* = 0.473, *p* < 0.001) or VEGF-A (*Pearson correlation r* = 0.574, *p* < 0.001) expression while CD68 expression was moderately correlated with VEGF-C (*Pearson correlation r* = 0.337, *p* < 0.001) expression in squamous cell carcinoma. There were also moderate correlations between CD163 and VEGF-A (*Pearson correlation r* = 0.356, *p* < 0.001) or VEGF-C (*Pearson correlation r* = 0.325, *p* < 0.001) expression in squamous cell carcinoma. Moreover, there were significant correlations between VEGF-A and VEGF-C in both adenocarcinoma (*Pearson correlation r* = 0.439, *p* < 0.001) and squamous cell carcinoma (*Pearson correlation r* = 0.574, *p* < 0.001) (Additional file 2: Fig. S2, S3).

### Survival analysis of TAMs, VEGF-A, and VEGF-C expression

**Fig. 3:**
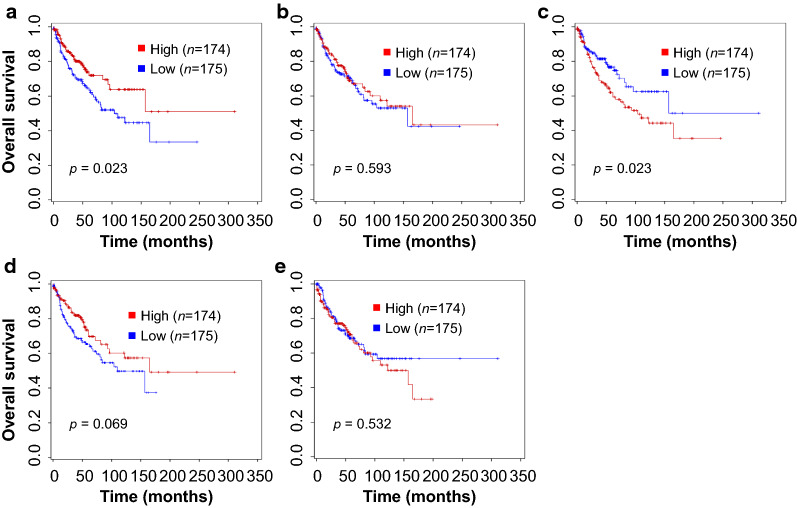
The Kaplan–Meier survival analysis of NSCLC patients. Patients expressing CD68 had better overall survival than patients who did not (OS rate, 78.2% *vs.* 68.6%, log rank *p* = 0.023) (**a**), while CD163 was not associated with patient survival (**b**). Patients with a high M2 ratio (CD163/CD68) had significantly shorter overall survival than patients with a low M2 ratio (OS rate, 68.4% *vs.* 78.3%, log rank *p* = 0.023) (**c**). There were no meaningful overall survival differences for VEGF-A (**d**) and VEGF-C (**e**) expression in NSCLC patients

We next examined the relationship between TAMs, VEGF-A, and VEGF-C expression and patient survival outcomes in 349 NSCLC patients with available overall survival data. NSCLC patients with high CD68 expression displayed significantly better overall survival (OS) (log-rank *p* = 0.023) than those with low CD68 expression (Fig. 3a). In contrast, a significant OS difference was observed between patients with a high M2 ratio (log-rank *p* = 0.023) and patients with a low M2 ratio (Fig. 3c). In subgroup analyses, adenocarcinoma NSCLC patients with high CD68 expression (log-rank *p* = 0.034) showed a significant survival advantage (Additional file 2: Fig. S4a), whereas adenocarcinoma NSCLC patients with high M2 ratio (log-rank *p* = 0.047) had poor survival (Additional file 2: Fig. S4c). However, CD68, CD163, VEGF-A, and VEGF-C expression, as well as M2 ratio, was not found to be associated with patient OS in squamous cell carcinomas (Additional file 2: Fig. S5).

**Fig. 4:**
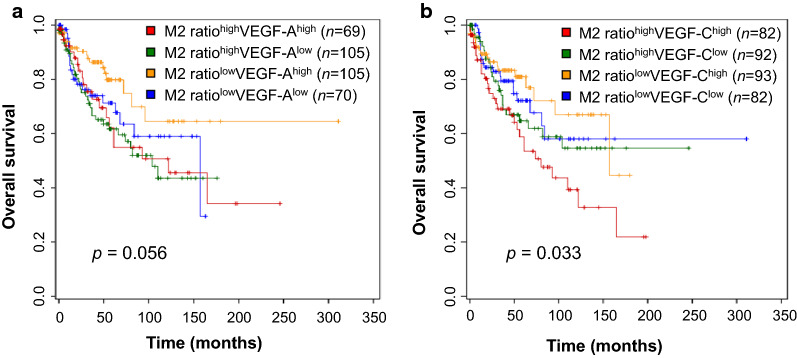
Survival analysis of NSCLC patients with M2 ratio expression according to angiogenesis (VEGF-A) or lymphangiogenesis (VEGF-C) marker expression in NSCLC patients. Dual expression of high M2 ratio and high VEGF-A (M2 ratio^high/^VEGF-A^high^) exhibited a tendency of worse overall survival (*p* = 0.056) (**a**). Survival times for patients with both high M2 ratio and high VEGF-C (M2 ratio^high^VEGF-C^high^) expression was significantly shorter (OS rate, 62.2%) than those for patients with M2 ratio^low^VEGF-C^low^ (OS rate, 75.6%), M2 ratio^low^VEGF-C^high^ (OS rate, 80.6%), and M2 ratio^high^VEGF-C^low^ (OS rate, 73.9%) (log rank *p* = 0.033) (**b**)

To investigate whether M2 ratio and VEGF family members have a combined effect on NSCLC prognosis, patients were divided into 4 groups according to M2 ratio and VEGF-A expression: M2 ratio^low^VEGF-A^low^, M2 ratio^low^VEGF-A^high^, M2 ratio^high^VEGF-A^low^, and M2 ratio^high^VEGF-A^high^. In pairwise comparisons, patients with M2 ratio^high^VEGF-A^high^ had significantly worse survival than those with M2 ratio^low^VEGF-A^high^ (*p* = 0.004), while there were no significant survival differences between M2 ratio^low^VEGF-A^low^ (*p* = 0.728) and M2 ratio^high^VEGF-A^low^ (*p* = 0.714). Kaplan–Meier plots revealed that patients with M2 ratio^high^VEGF-A^high^ had a tendency of worse overall survival, but this trend was not significant (log-rank *p* = 0.056, Fig. 4a). We next analyzed a potential correlation between prognosis and the combination of M2 ratio and VEGF-C expression in patients with NSCLC. In pairwise comparisons, patient survival in the M2 ratio^high^VEGF-C^high^ group was significantly worse than that for the M2 ratio^low^VEGF-C^low^ (log-rank *p* = 0.047) and M2 ratio^low^VEGF-C^high^ (log-rank *p* = 0.008) groups. However, there was no significant survival difference between M2 ratio^high^VEGF-C^high^ and M2 ratio^high^VEGF-C^low^ (log-rank *p* = 0.112) groups. Patients with M2 ratio^high^VEGF-C^high^ had the worst overall survival compared to patients with M2 ratio^low^VEGF-C^high^ (OS rate, 62.6% *vs.* 80.6%, log-rank *p* = 0.033, Fig. 4b).

In subgroup analyses, adenocarcinoma NSCLC patients with M2 ratio^high^VEGF-A^high^ had shorter survival time than those with M2 ratio^low^VEGF-A^high^ (OS rate, 72.7% *vs.* 93.3%, log-rank *p* = 0.027) (Additional file 2: Fig. S6a). Similarly, the survival times of patients with M2 ratio^high^VEGF-C^high^ were worse than those of patients with M2 ratio^low^VEGF-C^high^ (OS rate, 69.2% *vs.* 91.5%, log-rank *p* = 0.042) (Additional file 2: Fig. S6b). In pairwise comparisons, significant survival difference was observed between groups with M2 ratio^high^VEGF-A^high^ and M2 ratio^low^VEGF-A^high^ (*pairwise comparison*, *p* = 0.003). Similarly, there also significant difference of survival rate between groups M2 ratio^high^VEGF-C^high^ and with M2 ratio^low^VEGF-C^high^ (*pairwise comparison*, *p* = 0.004) expression in adenocarcinomas. However, there were no significant survival differences for combined M2 ratio and VEGF-A or VEGF-C expression in squamous cell carcinomas (Additional file 2: Fig. S7).

### Univariate and multivariate analyses for overall survival

Clinicopathological characteristics were associated with patient survival by univariate analysis (Table 2). High pT stage (*p* < 0.001), high pN stage (*p* < 0.001), high M2 ratio (*p* = 0.024), and dual M2 ratio^high^ and VEGF-C^high^ (*p* = 0.007) were associated with worse patient survival, while high CD68 expression correlated with good survival (*p* = 0.024). However, age, tumor grade, high CD163 expression, high VEGF-A expression, high VEGF-C expression, dual M2 ratio^high^ and VEGF-A^high^ were not associated with patient survival. Furthermore, Cox multivariate proportional hazard analysis revealed that high pT stage (hazard ratio 2.91 [95% CI 1.77–4.78], *p* < 0.001), high pN stage (hazard ratio 2.02 [95% CI 1.31–3.11], *p* = 0.002), high M2 ratio (hazard ratio 1.53 [95% CI 1.01–2.33], *p* = 0.046), and dual M2 ratio^high^ and VEGF-C^high^ expression (hazard ratio 2.01 [95% CI 1.28–3.16], *p* = 0.003) are independent prognosis factors for poor overall survival in NSCLC patients (Table 3).

**Table 2:** Univariate analysis of the association between prognostic variables and overall survival in NSCLC

Variables	Hazard ratio (95% CI)	*p* value
Age	1.01 (0.99–1.04)	0.267
Tumor grade	1.22 (0.93–1.60)	0.146
pT stage	3.57 (2.21–5.76)	< 0.001
pN stage	2.59 (1.72–3.91)	< 0.001
CD68^high^	0.62 (0.41–0.94)	0.024
CD163^high^	0.89 (0.60–1.35)	0.594
VEGF-A^high^	0.68 (0.45–1.03)	0.071
VEGF-C^high^	1.14 (0.76–1.71)	0.531
M2 ratio^high^	1.61 (1.07–2.44)	0.024
Dual M2 ratio^high^ & VEGF-A^high^	1.23 (0.74–2.02)	0.424
Dual M2 ratio^high^ & VEGF-C^high^	1.82 (1.18–2.80)	0.007

M2 ratio, CD163+/CD68+

*NSCLC *non-small cell lung cancer, *CI* confidence interval, *VEGF* vascular endothelial growth factor

**Table 3:** Multivariate analysis of the association between prognostic variables and overall survival in NSCLC

Variables	Hazard ratio (95% CI)	Se (Coef)	z	*p* value
pT stage	2.91 (1.77–4.78)	0.254	4.196	< 0.001
pN stage	2.02 (1.31–3.11)	0.221	3.171	0.002
M2 ratio^high^	1.53 (1.01–2.33)	0.214	1.992	0.046
Dual M2 ratio^high^ & VEGF-C^high^	2.01 (1.28–3.16)	0.231	3.028	0.003

M2 ratio, CD163+/CD68+

*NSCLC *non-small cell lung cancer, *CI* confidence interval, *VEGF* vascular endothelial growth factor

### Effect of macrophage polarization on angiogenesis and lymphangiogenesis

**Fig. 5:**
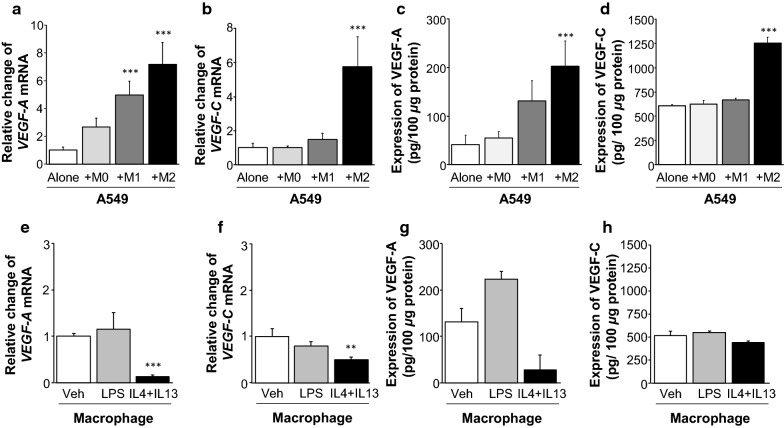
Effect of macrophage polarization on NSCLC cell angiogenesis and lymphangiogenesis. A549 cells were co-cultured with M0 macrophages, M1 macrophages, polarized by LPS, or M2 macrophages, polarized by IL4 + IL13. After 48 h of co-culture, total RNA and protein were extracted from the cells as indicated. Gene expression was assessed with quantitative real-time PCR (**a**, **b**), and protein expression was quantitated using specific ELISA kits (**c**, **d**). *VEGF-A* and *VEGF-C* mRNA levels increased in M2 macrophages significantly. However, M1 macrophages elevated only *VEGF-A* mRNA, not *VEGF-C,* in A549 cells. In order to assess the levels of *VEGF-A* and *VEGF-C* mRNA in polarized macrophages, the macrophages were collected after 72 h of culturing with LPS or IL4 + IL13, total RNA and proteins were extracted, and gene and protein expression were assessed with quantitative real-time PCR (**e**, **f**) and ELISA (**g**, **h**) as well. The mRNA levels of *VEGF-A* and *VEGF-C* were significantly decreased in M2 macrophages polarized by IL4 + IL13, compared to M1 macrophages polarized by LPS or M0 macrophages cultured with vehicles. Error bars represent S.D; *p < 0.05, **p < 0.01, ***p < 0.001. Asterisk represents the significance compared to A549 alone or macrophage with vehicle

In order to investigate the effect of M1 and M2 macrophages on VEGF-A and VEGF-C expression in NSCLC, macrophages polarized toward M1 type (pro-inflammatory) or M2 type (anti-inflammatory) were co-cultured with A549 cells, a human NSCLC cell line. Human monocytic THP-1 cells were differentiated into macrophages (unpolarized M0 macrophages) by PMA treatment for 24 h, and these macrophages were seeded into transwell inserts. M0 THP-1 macrophages were polarized toward M1 type by 24 h incubation with LPS (10 ng/ml), or M2 type with recombinant human IL4 + IL13 (each of 20 ng/ml) in transwell inserts. Polarized M1, M2, and unpolarized M0 macrophages were washed 3 times with PBS, and co-cultured with A549 cells. VEGF-A and VEGF-C protein levels in cell lysate were assessed by ELISA, and mRNA levels in THP-1 macrophages and A549 cells at the time of co-culture were measured by quantitative real-time PCR. As shown in Fig. 5a–d, there were no significant changes in mRNA and protein expression of VEGF-A and VEGF-C in A549 cells co-cultured with M0 macrophages. A549 cells co-cultured with M2 macrophages exhibited significantly elevated VEGF-A and VEGF-C protein and mRNA levels (*p* < 0.001) compared to those of A549 cells alone. M1 macrophages induced mRNA expression of *VEGF-A* significantly in A549 cells (*p* = 0.004), but did not affect VEGF-A protein levels. Interestingly, M1 macrophages did not affect VEGF-C protein and mRNA expression in A549 cells. Co-culturing with M2 macrophages drastically increased the levels of *VEGF-A* and *VEGF-C* mRNA in A549 cells, compared to co-culturing with M1 macrophages. The protein and mRNA expression levels of both molecules were assessed in THP-1 macrophages (Fig. 5e–h) as well. VEGF-A protein and mRNA expression did not significantly differ between M1 macrophages treated with LPS and unpolarized M0 unpolarized macrophages (with vehicle). Contrastingly, VEGF-C protein and mRNA levels were decreased in M2 macrophages treated with IL4 + IL13 compared to M0, unpolarized macrophages.

## Discussion

The management of advanced NSCLC has significantly improved in recent years with the advent of molecular-targeted therapies [27, 28]. Nonetheless, the prognosis of advanced NSCLC remains poor and the outcomes for NSCLC patients have improved finitely during the past decades. Recently, immunotherapies using immune checkpoint inhibitors have exhibited their superiority over chemotherapy, especially in advanced stage NSCLC patients [5, 29, 30]. However, only a limited number of patients achieved significant improvement in overall survival and progression-free survival amongst the unscreened and immunotherapy-treated patients. In addition, immunotherapy has limitations including many side effects and expensive treatment costs. It is especially important to find a reliable biomarker that can be used to precisely screen NSCLC patients for immunotherapy. In this study, we confirmed that patients with high M1 macrophage expression had significantly better overall survival compared to patients with low infiltration of M1 macrophages, while patients with high M2 ratio (CD163+/CD68+) had significantly worse overall survival compared to that of patients with low M2 ratio. Furthermore, we found a potential linkage between TAMs, angiogenesis and lymphangiogenesis. Notably, the combination of high M2 ratio and high VEGF-C expression was an independent prognostic factor for poor overall survival in NSCLC patients.

The TME, which consists of various cells and extracellular components, has been spotlighted for not only playing a pivotal role during tumor initiation, progression and metastasis, but also being highly associated with tumor relapse after conventional anticancer therapies. TAMs are key components of the TME and can have functionally distinct characteristics in response to environmental cues [31, 32]. They are categorized into two subsets: classically activated (M1) or alternately activated (M2). M1 macrophages inhibit tumor growth by producing reactive oxygen intermediates, reactive nitrogen intermediates, and tumor necrosis factor alpha (TNFα), whereas M2 macrophages promote tumor growth and metastasis by secreting matrix-degrade enzymes, angiogenic factors and immunosuppressive cytokines/chemokines [33]. Thus, the quantitation of TAM expression can be an invaluable clinical indicator for managing patients with NSCLC. Ma et al. [34] and Rakaee et al. [35] have previously reported that a high density of M1 macrophages in tumor islets and tumor stroma is associated with favorable patient survival outcomes. Likewise, our study results showed that high CD68 expression in tumor stroma is associated with good prognosis. On the other hand, Cao et al. found no correlation between CD68 density in the tumor interstitial region and overall survival of NSCLC patients [36]. These controversial data may be explained by the unique characteristics of TAMs, which have dynamic and heterogenous phenotypes in response to the local TME. In addition, CD68 is a relatively nonspecific marker, necessitating the development of a specific marker for M1 macrophages. Similarly, reports on the prognostic value of M2 macrophages in NSCLC are inconsistent. CD163 is a specific marker for M2 macrophages and has been used for immunohistochemistry via single or ratio assessment (CD163/CD68) [37]. Previous studies have shown that high levels of M2 macrophages in tumor islets and stroma are positively associated with negative outcomes in NSCLC patients, while others found no correlation between M2 macrophages and clinical outcomes of NSCLC patients [34]. Prior studies demonstrated that IHC interpreted via digital image analysis allows better predictions of prognostic relevance than manual visual scoring [38, 39]. We have also previously confirmed that digital image analysis resulted in better clinical value than traditional manual scoring [40]. Moreover, we have also demonstrated that ratio-based biomarkers can provide enhanced prognostic value over assessment of individual biomarkers [41, 42]. This approach requires continuous quantitative values from digital image analysis. We found that TAMs were mostly distributed in the tumor stroma, which corroborates previous data [43]. Thus, we assessed TAM expression by combining immunohistochemistry and quantitative image analysis to study the tumor stroma. High M2 ratio (CD163+/CD68+) was associated with poor prognosis in NSCLC, but there was no meaningful clinical value from the M2 macrophage assessment using only CD163. Notably, high M2 ratio was an independent prognostic factor for poor overall survival in NSCLC patients. In contrast, Rakaee et al. reported that high M2 ratio (CD204+/CD68+) was an independent prognostic factor for good disease specific survival in NSCLC patients [35]. This inconsistency may partially be explained by the lack of a standard algorithm for TAMs, different IHC methodology, or differences in the studies’ patient populations.

Previous studies suggested that the VEGF-A/vascular endothelial growth factor receptor-2 (VEGFR-2) axis is the major pathway for angiogenesis [44], while the VEGF-C/VEGF-D/ vascular endothelial growth factor receptor-3 (VEGFR-3) axis is involved in lymphangiogenesis of cancer [45]. VEGF-A is an endogenous agonist for VEGFR-2 and its signaling is targeted through neutralizing circulating VEGF-A using bevacizumab [46], or inhibiting downstream signaling pathways [47]. On the other hand, VEGF-C overexpression promotes nodal and distant organ metastasis [48], while VEGF-C knockdown inhibits these properties [49]. We previously demonstrated that SCP3 expression is closely associated with VEGF-C and VEGF-D expression, and potentially linked with lymphangiogenesis in NSCLC patients [40]. Given that VEGF-A and VEGF-C has been implicated in angiogenesis and lymphangiogenesis in cancer development, many studies have investigated the prognostic value of VEGF-A and VEGF-C in NSCLC patient tissues. However, the prognostic value of VEGF-A and VEGF-C is still controversial. VEGF-A has been reported to be associated with survival time [50, 51], but other reports conflict with these results [52, 53]. A similar finding for the clinical value of VEGF-C in NSCLC has also been previously reported. VEGF-C has been shown to be linked with lymph node metastasis in NSCLC [54, 55]. Jiang et al., showed that VEGF-C expression is associated with poor prognosis for NSCLC patients, but not with clinical outcomes for patients with lung adenocarcinoma, via meta-analysis of 1988 patients aggregated from 16 trials [56]. In contrast, prior studies indicated there is no significant correlation between VEGF-C expression and lymph node metastasis in NSCLC. Interestingly, *VEGF-C* mRNA expression in patients with lymph node metastasis was lower compared to that of patients without metastasis [57]. Previous studies also reported that there is no correlation between VEGF-C expression and lymph node metastasis [58, 59]. In this study, we also observed that there was no meaningful association between VEGF-A and prognostic value in NSCLC. Similarly, VEGF-C expression did not correlate with lymph node metastasis. The complex environment of VEGFs and VEGFRs during NSCLC development might be one of the reasons for these inconsistent conclusions. In this study, we demonstrated that the analysis of macrophage subtypes could improve prognosis prediction for NSCLC patients. Moreover, we found that accumulation of M2 TAMs is positively associated with levels of VEGF-A and VEGF-C in NSCLC. Further studies are needed to define whether these combinational markers will help screen NSCLC patients for immunotherapy.

TAMs generally acquire a M2-like phenotype [43] to help promote tumor growth and metastasis [60, 61]. It is thought that TAMs are regulated by an angiogenic switch, which is a critical step in the transition to malignancy. Lin et al. demonstrated that inhibition of macrophage infiltration in tumors delays the angiogenic switch and malignant transition in a mouse model of breast cancer [62]. This study suggests that the angiogenic switch does not occur in the absence of macrophages. Moreover, during the angiogenetic switch, macrophages promote blood vessel maturation and vascular permeability by VEGF secretion [63]. Recently, Alishekevitz et al. demonstrated that TAMs promote lymphangiogenesis via the VEGF-C/VEGFR-3 pathway [64]. However, the detailed molecular mechanism behind the association of TAMs and angiogenesis or lymphangiogenesis is not fully understood. To understand the dynamics of polarized TAMs in the context of lymphangiogenesis via VEGF-A and -C in NSCLC, A549 cells were co-cultured with THP-1 macrophages polarized by LPS (for M1 type) or interleukin 4 (IL4)/interleukin 13 (IL13) (for M2 type) treatment. Interestingly, VEGF-A and -C protein levels remained unchanged in M1 and M2 macrophages, while mRNA levels of both molecules were significantly decreased in M2 macrophages. However, M2 macrophages provoked a significant increase in VEGF-A and -C proteins and mRNA in A549 cells, while M1 macrophages only increased *VEGF-A* mRNA. These data suggest that accumulated M2 TAMs can stimulate the production of VEGF-A and -C, which promote angiogenesis and lymphangiogenesis. VEGF-A and -C mediated angiogenesis and lymphangiogenesis enhances the potential of NSCLC metastasis. Since the M2 macrophage and A549 co-cultures did not allow cell–cell interaction, secretory molecules from M2 macrophages were involved in this interaction. Therefore, further studies should be performed to find potential stimulatory molecules that are secreted to aid the production of VEGF-A and -C in NSCLC, and ultimately develop anticancer therapeutics to target this interaction.

## Conclusions

CD68 and CD163 expression in tumor stroma were positively correlated with VEGF-A and VEGF-C in NSCLC patients’ tissues. High M2 ratio (CD163+/CD68+) in the tumor stroma is a potential marker for predicting malignant clinical outcomes in NSCLC patients, and consideration of M2 ratio and VEGF-C expression in combination may enhance the accuracy of prognostic prediction. Furthermore, our findings suggest that increased M2 TAMs can promote VEGF-A and -C expression in NSCLC cells, which contribute to angiogenesis and lymphangiogenesis within the tumor site. Further studies are warranted to explore the detailed molecular mechanisms of the crosstalk between M2 macrophages and VEGFs in NSCLC.

## Supplementary information

**Additional file 1: Table S1.** Clinicopathological characteristics of patients with NSCLC. **Table S2.** Association between clinicopathological factors and combination of M2 ratio and VEGF-A expression in NSCLC. **Table S3.** Association between clinicopathological factors and combination of M2 ratio and VEGF-C expression in NSCLC. **Table S4.** Association between clinicopathological parameters and CD68, CD163, VEGF-A, or VEGF-C expression as well as M2 ratio in NSCLC patients with squamous cell carcinomas. **Table S5.** Association between clinicopathological parameters and CD68, CD163, VEGF-A, or VEGF-C expression as well as M2 ratio in NSCLC patients with adenocarcinomas

**Additional file 2: Fig. S1** Association between tumor-associated macrophage and VEGFs in human non-small cell lung cancer (NSCLC). Correlation between tumor-associated macrophage and VEGF-A (**a**) and VEGF-C (**b**). **Fig. S2** Correlation among tumor-associated macrophage-, angiogenesis- and lymphangiogenesis-related markers in patients with adenocarcinoma NSCLC. CD68 expression positively correlated with CD163 (**a**), VEGF-A (**b**), and VEGF-C (**c**) expression. CD163 expression showed a significant positive correlation with VEGF-A (**d**), but a weak correlation with VEGF-C (**e**) expression. There is a positive correlation between VEGF-A and VEGF-C (**f**). **Fig. S3** Correlation among tumor-associated macrophage-, angiogenesis- and lymphangiogenesis-related markers in patients with squamous cell carcinoma NSCLC. CD68 expression positively correlated with CD163 (**a**), VEGF-A (**b**), and VEGF-C (**c**) expression. CD163 expression showed a moderate correlation with VEGF-A (**d**) and VEGF-C (**e**) expression. There is a significant positive correlation between VEGF-A and VEGF-C (**f**). **Fig. S4** Kaplan–Meier survival curves for tumor-associated macrophage-, angiogenesis- and lymphangiogenesis-related markers in NSCLC patients with adenocarcinoma. Patients expressing CD68 had better overall survival than patients who did not (OS rate, 85.9% *vs.* 75.7%, log rank *p* = 0.034) (**a**), while CD163 was not associated with patient survival (*p* = 0.470) (**b**). Patients with a high M2 ratio (CD163/CD68) had significantly shorter overall survival than patients with a low M2 ratio (OS rate, 76.0% *vs.* 84.5%, log rank *p* = 0.047) (**c**). There were no meaningful overall survival differences for VEGF-A (*p* = 0.092) (**d**) and VEGF-C (*p* = 0.899) (**e**) expression in NSCLC patients. **Fig. S5** Kaplan–Meier survival curves for tumor-associated macrophage-, angiogenesis- and lymphangiogenesis-related markers in NSCLC patients with squamous cell carcinoma. There were no meaningful overall survival differences for CD 68 (*p* = 0.153) (**a**), CD163 (*p* = 0.449) (**b**), M2 ratio (*p* = 0.682) (**c**), VEGF-A (*p* = 0.091) (**d**) and VEGF-C (*p* = 0.670) (**e**) expression in NSCLC patients with squamous cell carcinoma. **Fig. S6** Survival analysis of NSCLC patients with M2 ratio expression according to angiogenesis (VEGF-A) or lymphangiogenesis (VEGF-C) marker expression in NSCLC patients with adenocarcinoma. Survival differences were observed among 4 NSCLC patient groups classified according to their M2 ratio and VEGF-A expression (log rank *p* = 0.027) (**a**). A significant difference of survival rate was found among 4 NSCLC patient groups classified according to their M2 ratio and VEGF-C expression (log rank *p* = 0.042) (**b**). **Fig. S7** Survival analysis of NSCLC patients with M2 ratio expression according to angiogenesis (VEGF-A) or lymphangiogenesis (VEGF-C) marker expression in NSCLC patients with squamous cell carcinoma. There were no meaningful overall survival differences for combination M2 ratio and VEGF-A (*p* = 0.387) (**a**) or VEGF-C (*p* = 0.551) (**b**).

## Data Availability

The datasets used and/or analyzed for the current study are available upon reasonable request to the corresponding author.

## Abbreviations

TME: Tumor microenvironment
TAM: Tumor associated macrophage
NSCLC: Non-small cell lung cancer
TNM: Tumor, node, metastasis
IASLC: International Association for the Study of Lung Cancer
AJCC: American Joint Committee on Cancer
PD-L1: Programmed death ligand 1
TMB: Tumor mutation burden
TIL: Tumor-infiltrating lymphocytes
MSI: Microsatellite instability
VEGF: Vascular endothelial growth factor
WHO: World Health Organization
TMA: Tissue microarray
FFPE: Formalin-fixed, paraffin-embedded
H&E: Hematoxylin and eosin
DAB: 3–3′-Diaminobenzidine
HRP: Horseradish peroxidase
AP: Alkaline phosphatase
DIA: Digital image analysis
PMA: Phorbol 12-myristate 13-acetate
LPS: Lipopolysaccharide
PCR: Polymerase chain reaction
ELISA: Enzyme-linked immunosorbent assay
HR: Hazard ratio
CI: Confidence interval
TNFα: Tumor necrosis factor alpha
VEGFR-2: Vascular endothelial growth factor receptor-2
VEGFR-3: Vascular endothelial growth factor receptor-3
IL4: Interleukin 4
IL13: Interleukin 13
