# Supplementary material for: Tumor-associated macrophage, angiogenesis and lymphangiogenesis markers predict prognosis of non-small cell lung cancer patients
Source: J Transl Med. 2020 Nov 23;18:443. doi: 10.1186/s12967-020-02618-z (PMC7686699; doi:10.1186/s12967-020-02618-z)
Supplement: Supplementary file 1 — Additional file 1: Table S1. Clinicopathological characteristics of patients with NSCLC. Table S2. Association between clinicopathological factors and combination of M2 ratio and VEGF-A expression in NSCLC. Table S3. Association between clinicopathological factors and combination of M2 ratio and VEGF-C expression in NSCLC. Table S4. Association between clinicopathological parameters and CD68, CD163, VEGF-A, or VEGF-C expression as well as M2 ratio in NSCLC patients with squamous cell carcinomas. Table S5. Association between clinicopathological parameters and CD68, CD163, VEGF-A, or VEGF-C expression as well as M2 ratio in NSCLC patients with adenocarcinomas [file 12967_2020_2618_MOESM1_ESM.docx]

**Supplementary Table S1. Clinicopathological characteristics of patients with NSCLC**

| **Category (*n* = 349)** | **No. (%)** |
| --- | --- |
| Age |  |
| < 60 years | 75 (21.5) |
| ≥ 60 years | 274 (78.5) |
| Sex |  |
| Male | 241 (69.1) |
| Female | 108 (30.9) |
| Tumor type |  |
| SqCC | 135 (38.7) |
| AD | 210 (60.2) |
| ADSq | 4 (1.1) |
| Grade |  |
| Well | 125 (35.8) |
| Moderate | 158 (45.3) |
| Poor | 66 (18.9) |
| pT stage |  |
| T1 | 155 (44.4) |
| T2 | 148 (42.4) |
| T3 | 43 (12.3) |
| T4 | 3 (0.9) |
| pN stage |  |
| pN0 | 247 (70.8) |
| pN1 | 54 (15.4) |
| pN2 | 46 (13.2) |
| pN3 | 2 (0.6) |
| Stage group |  |
| I | 218 (62.5) |
| II | 72 (20.6) |
| III | 52 (14.9) |
| IV | 7 (2.0) |
| Status |  |
| Alive | 256 (73.4) |
| Expire | 93 (26.6) |

NSCLC, non-small cell lung cancer; AD, adenocarcinoma; SqCC, squamous cell carcinoma; ADSq, adenosquamous carcinoma

**Supplementary Table S2. Association between clinicopathological factors and combination of M2 ratio and VEGF-A expression in NSCLC**

| **Category**  (*n* = 349) | **Combination M2 ratio and VEGF-A expression** | | | | | | | | |
| --- | --- | --- | --- | --- | --- | --- | --- | --- | --- |
|  | Low & Low  No. (%) |  | Low & High  No. (%) |  | High & Low  No. (%) |  | High & High  No. (%) |  | ***p*** value |
| Total | 70 (20.0) |  | 105 (30.1) |  | 105 (30.1) |  | 69 (19.8) |  |  |
| Age |  |  |  |  |  |  |  |  | 0.822 |
| < 60 years | 14 (20.0) |  | 25 (23.8) |  | 20 (19.0) |  | 16 (23.2) |  |  |
| ≥ 60 years | 56 (80.0) |  | 80 (76.2) |  | 85 (81.0) |  | 53 (76.8) |  |  |
| Sex |  |  |  |  |  |  |  |  | 0.022 |
| Male | 42 (60.0) |  | 68 (64.8) |  | 74 (70.5) |  | 57 (82.6) |  |  |
| Female | 28 (40.0) |  | 37 (35.2) |  | 31 (29.5) |  | 12 (17.4) |  |  |
| Tumor type |  |  |  |  |  |  |  |  | 0.075 |
| SqCC | 19 (27.2) |  | 44 (41.9) |  | 36 (34.3) |  | 36 (52.2) |  |  |
| AD | 50 (71.4) |  | 60 (57.1) |  | 67 (63.8) |  | 33 (47.8) |  |  |
| ADSq | 1 (1.4) |  | 1 (1.0) |  | 2 (1.9) |  | 0 (0.0) |  |  |
| Grade |  |  |  |  |  |  |  |  | 0.741 |
| Well | 26 (37.1) |  | 31 (29.5) |  | 41 (39.1) |  | 27 (39.1) |  |  |
| Moderate | 33 (47.1) |  | 50 (47.6) |  | 46 (43.8) |  | 29 (42.0) |  |  |
| Poor | 11 (15.8) |  | 24 (22.9) |  | 18 (17.1) |  | 13 (18.9) |  |  |
| pT stage |  |  |  |  |  |  |  |  | 0.742 |
| T1-2 | 62 (88.6) |  | 90 (85.7) |  | 89 (84.8) |  | 62 (89.9) |  |  |
| T3-4 | 8 (11.4) |  | 15 (14.3) |  | 16 (15.2) |  | 7 (10.1) |  |  |
| pN stage |  |  |  |  |  |  |  |  | 0.475 |
| 0 | 47 (67.1) |  | 80 (76.2) |  | 74 (70.5) |  | 46 (66.7) |  |  |
| 1-3 | 23 (32.9) |  | 25 (23.8) |  | 31 (29.5) |  | 23 (33.3) |  |  |
| Stage group |  |  |  |  |  |  |  |  | 0.101 |
| I | 40 (57.2) |  | 70 (66.7) |  | 66 (62.8) |  | 42 (60.9) |  |  |
| II | 18 (25.7) |  | 20 (19.0) |  | 16 (15.2) |  | 18 (26.1) |  |  |
| III | 8 (11.4) |  | 13 (12.4) |  | 22 (21.0) |  | 9 (13.0) |  |  |
| IV | 4 (5.7) |  | 2 (1.9) |  | 1 (1.0) |  | 0 |  |  |

NSCLC, non-small cell lung cancer; AD, adenocarcinoma; SqCC, squamous cell carcinoma; ADSq, adenosquamous carcinoma; VEGF, vascular endothelial growth factor

**Supplementary Table S3.** **Association between clinicopathological factors and combination of M2 ratio and VEGF-C expression in NSCLC**

| **Category**  (*n* = 349) | **Combination M2 ratio and VEGF-C expression** | | | | | | | | |
| --- | --- | --- | --- | --- | --- | --- | --- | --- | --- |
|  | Low & Low  No. (%) |  | Low & High  No. (%) |  | High & Low  No. (%) |  | High & High  No. (%) |  | ***p*** value |
| Total | 82 (23.5) |  | 93 (26.6) |  | 92 (26.4) |  | 82 (23.5) |  |  |
| Age |  |  |  |  |  |  |  |  | 0.475 |
| < 60 years | 17 (20.7) |  | 25 (23.7) |  | 30 (32.6) |  | 13 (15.9) |  |  |
| ≥ 60 years | 65 (79.3) |  | 71 (76.3) |  | 62 (67.4) |  | 69 (84.1) |  |  |
| Sex |  |  |  |  |  |  |  |  | <0.001 |
| Male | 43 (52.4) |  | 67 (72.0) |  | 62 (67.4) |  | 69 (84.1) |  |  |
| Female | 39 (47.6) |  | 26 (28.0) |  | 30 (32.6) |  | 13 (15.9) |  |  |
| Tumor type |  |  |  |  |  |  |  |  | <0.001 |
| SqCC | 17 (20.7) |  | 46 (49.5) |  | 29 (31.5) |  | 43 (52.4) |  |  |
| AD | 63 (76.8) |  | 47 (50.5) |  | 61 (66.3) |  | 39 (47.6) |  |  |
| ADSq | 2 (2.5) |  | 0 |  | 2 (2.2) |  | 0 |  |  |
| Grade |  |  |  |  |  |  |  |  | 0.010 |
| Well | 37 (45.1) |  | 20 (21.5) |  | 41 (44.6) |  | 27 (32.9) |  |  |
| Moderate | 30 (36.6) |  | 53 (57.0) |  | 33 (35.9) |  | 42 (51.2) |  |  |
| Poor | 15 (18.3) |  | 20 (21.5) |  | 18 (19.5) |  | 13 (15.9) |  |  |
| pT stage |  |  |  |  |  |  |  |  | 0.988 |
| T1-2 | 72 (87.8) |  | 80 (86.0) |  | 80 (87.0) |  | 71 (86.6) |  |  |
| T3-4 | 10 (12.2) |  | 13 (14.0) |  | 12 (13.0) |  | 11 (13.4) |  |  |
| pN stage |  |  |  |  |  |  |  |  | 0.453 |
| 0 | 63 (76.8) |  | 64 (68.8) |  | 66 (71.7) |  | 54 (65.9) |  |  |
| 1-3 | 19 (23.2) |  | 29 (31.2) |  | 26 (28.3) |  | 28 (34.1) |  |  |
| Stage group |  |  |  |  |  |  |  |  | 0.136 |
| I | 57 (69.5) |  | 53 (57.0) |  | 61 (66.3) |  | 47 (57.3) |  |  |
| II | 12 (14.6) |  | 26 (28.0) |  | 15 (16.3) |  | 19 (23.2) |  |  |
| III | 11 (13.4) |  | 10 (10.7) |  | 15 (16.3) |  | 16 (19.5) |  |  |
| IV | 2 (2.5) |  | 4 (4.3) |  | 1 (1.1) |  | 0 |  |  |

NSCLC, non-small cell lung cancer; AD, adenocarcinoma; SqCC, squamous cell carcinoma; ADSq, adenosquamous carcinoma; VEGF, vascular endothelial growth factor

**Supplementary Table S4. Association between clinicopathological parameters and CD68, CD163, VEGF-A, or VEGF-C expression as well as M2 ratio in NSCLC patients with squamous cell carcinoma**

| **Category**  (*n* = 135) | **CD68** | | |  | **CD163** | | |  | **CD163+/CD68+ (M2 ratio)** | | |  | **VEGF-A** | | |  | **VEGF-C** | | |
| --- | --- | --- | --- | --- | --- | --- | --- | --- | --- | --- | --- | --- | --- | --- | --- | --- | --- | --- | --- |
|  | High  *n* (%) | Low  *n* (%) | ***p*** |  | High  *n* (%) | Low  *n* (%) | ***p*** |  | High  *n* (%) | Low  *n* (%) | ***p*** |  | High  *n* (%) | Low  *n* (%) | ***p*** |  | High  *n* (%) | Low  *n* (%) | ***p*** |
| Total | 74 (54.8) | 61 (45.2) |  |  | 79 (58.5) | 56 (41.5) |  |  | 72 (53.3) | 63 (46.7) |  |  | 80 (59.3) | 55 (40.7) |  |  | 89 (65.9) | 46 (34.1) |  |
| Age |  |  | 0.916 |  |  |  | 0.985 |  |  |  | 1.000 |  |  |  | 0.071 |  |  |  | 1.000 |
| < 60 | 12 (16.2) | 11 (18.0) |  |  | 14 (17.7) | 9 (16.1) |  |  | 12 (16.7) | 11 (17.5) |  |  | 18 (22.5) | 5 (9.1) |  |  | 15 (16.9) | 8 (17.4) |  |
| ≥ 60 | 62 (83.8) | 50 (82.0) |  |  | 65 (82.3) | 47 (83.9) |  |  | 60 (83.3) | 52 (82.5) |  |  | 62 (77.5) | 50 (90.9) |  |  | 74 (83.1) | 38 (82.6) |  |
| Sex |  |  | 0.309 |  |  |  | 0.402 |  |  |  | 0.802 |  |  |  | 1.000 |  |  |  | 0.688 |
| Male | 69 (93.2) | 60 (98.4) |  |  | 74 (93.7) | 55 (98.2) |  |  | 68 (94.4) | 61 (96.8) |  |  | 76 (95.0) | 53 (96.4) |  |  | 86 (96.6) | 43 (93.5) |  |
| Female | 5 (6.8) | 1 (1.6) |  |  | 5 (6.3) | 1 (1.8) |  |  | 4 (5.6) | 2 (3.2) |  |  | 4 (5.0) | 2 (3.6) |  |  | 3 (3.4) | 3 (6.5) |  |
| Grade |  |  | 0.064 |  |  |  | 0.710 |  |  |  | 0.471 |  |  |  | 0.913 |  |  |  | 0.029 |
| well | 7 (9.4) | 11 (18.0) |  |  | 9 (11.4) | 9 (16.1) |  |  | 12 (16.7) | 6 (9.5) |  |  | 10 (12.4) | 8 (14.5) |  |  | 7 (7.9) | 11 (23.9) |  |
| moderate | 48 (64.9) | 43 (70.5) |  |  | 55 (69.6) | 36 (64.3) |  |  | 47 (65.3) | 44 (69.9) |  |  | 55 (68.8) | 36 (65.5) |  |  | 65 (73.0) | 26 (56.5) |  |
| poor | 19 (25.7) | 7 (11.5) |  |  | 15 (19.0) | 11 (19.6) |  |  | 13 (18.0) | 13 (20.6) |  |  | 15 (18.8) | 11 (20.0) |  |  | 17 (19.1) | 9 (19.6) |  |
| pT stage |  |  | 1.000 |  |  |  | 0.532 |  |  |  | 1.000 |  |  |  | 0.472 |  |  |  | 1.000 |
| T1-2 | 58 (78.4) | 48 (78.7) |  |  | 64 (81.0) | 42 (75.0) |  |  | 57 (79.2) | 49 (77.8) |  |  | 65 (81.2) | 41 (74.5) |  |  | 70 (78.7) | 36 (78.3) |  |
| T3-4 | 16 (21.6) | 13 (21.3) |  |  | 15 (19.0) | 14 (25.0) |  |  | 15 (20.8) | 14 (22.2) |  |  | 15 (18.8) | 14 (25.5) |  |  | 19 (21.3) | 10 (21.7) |  |
| pN stage |  |  | 0.281 |  |  |  | 0.953 |  |  |  | 0.285 |  |  |  | 1.000 |  |  |  | 0.391 |
| 0 | 57 (77.0) | 41 (67.2) |  |  | 58 (73.4) | 40 (71.4) |  |  | 49 (68.1) | 49 (77.8) |  |  | 58 (72.5) | 40 (72.7) |  |  | 62 (69.7) | 36 (78.3) |  |
| 1-3 | 17 (23.0) | 20 (32.8) |  |  | 21 (26.6) | 16 (28.6) |  |  | 23 (31.9) | 14 (22.2) |  |  | 22 (27.5) | 15 (27.3) |  |  | 27 (30.3) | 10 (21.7) |  |
| Stage |  |  | 0.657 |  |  |  | 0.670 |  |  |  | 0.576 |  |  |  | 0.224 |  |  |  | 0.602 |
| I | 45 (60.8) | 33 (54.1) |  |  | 47 (59.5) | 31 (55.4) |  |  | 40 (55.6) | 38 (60.3) |  |  | 47 (58.8) | 31 (56.4) |  |  | 49 (55.0) | 29 (63.1) |  |
| II | 21 (28.4) | 21 (34.4) |  |  | 24 (30.4) | 18 (32.1) |  |  | 23 (31.9) | 19 (30.2) |  |  | 27 (33.8) | 15 (27.3) |  |  | 28 (31.5) | 14 (30.4) |  |
| III | 7 (9.4) | 7 (11.5) |  |  | 8 (10.1) | 6 (10.7) |  |  | 9 (12.5) | 5 (7.9) |  |  | 5 (6.2) | 9 (16.3) |  |  | 11 (12.4) | 3 (6.5) |  |
| IV | 1 (1.4) | 0 |  |  | 0 | 1 (1.8) |  |  | 0 | 1 (1.6) |  |  | 1 (1.2) | 0 |  |  | 1 (1.1) | 0 |  |

NSCLC, non-small cell lung cancer; AD, adenocarcinoma; SqCC, squamous cell carcinoma; ADSq, adenosquamous carcinoma; VEGF, vascular endothelial growth factor

**Supplementary Table S5. Association between clinicopathological parameters and CD68, CD163, VEGF-A, or VEGF-C expression as well as M2 ratio in NSCLC patients with adenocarcinoma**

| **Category**  (*n* = 210) | **CD68** | | |  | **CD163** | | |  | **CD163+/CD68+ (M2 ratio)** | | |  | **VEGF-A** | | |  | **VEGF-C** | | |
| --- | --- | --- | --- | --- | --- | --- | --- | --- | --- | --- | --- | --- | --- | --- | --- | --- | --- | --- | --- |
|  | High  *n* (%) | Low  *n* (%) | ***p*** |  | High  *n* (%) | Low  *n* (%) | ***p*** |  | High  *n* (%) | Low  *n* (%) | ***p*** |  | High  *n* (%) | Low  *n* (%) | ***p*** |  | High  *n* (%) | Low  *n* (%) | ***p*** |
| Total | 99 (47.1) | 111 (52.9) |  |  | 94 (44.8) | 116 (55.2) |  |  | 100 (47.6) | 110 (52.4) |  |  | 93 (44.3) | 117 (55.7) |  |  | 86 (41.0) | 124 (59.0) |  |
| Age |  |  | 0.982 |  |  |  | 0.095 |  |  |  | 0.920 |  |  |  | 1.000 |  |  |  | 1.000 |
| < 60 | 23 (23.2) | 27 (24.3) |  |  | 28 (29.8) | 22 (19.0) |  |  | 23 (23.0) | 27 (24.5) |  |  | 22 (23.7) | 28 (23.9) |  |  | 20 (23.3) | 30 (24.2) |  |
| ≥ 60 | 76 (76.8) | 84 (75.7) |  |  | 66 (70.2) | 94 (81.0) |  |  | 77 (77.0) | 83 (75.5) |  |  | 71 (76.3) | 89 (76.1) |  |  | 66 (76.7) | 94 (75.8) |  |
| Sex |  |  | 0.806 |  |  |  | 0.936 |  |  |  | 0.008 |  |  |  | 1.000 |  |  |  | 0.172 |
| Male | 50 (50.5) | 59 (53.2) |  |  | 48 (51.1) | 61 (52.6) |  |  | 62 (62.0) | 47 (42.7) |  |  | 48 (51.6) | 56 (47.9) |  |  | 50 (58.1) | 59 (47.6) |  |
| Female | 49 (49.5) | 52 (46.8) |  |  | 46 (48.9) | 55 (47.4) |  |  | 38 (38.0) | 63 (57.3) |  |  | 45 (48.4) | 61 (52.1) |  |  | 36 (41.9) | 65 (52.4) |  |
| Grade |  |  | 0.153 |  |  |  | 0.771 |  |  |  | 0.291 |  |  |  | 0.104 |  |  |  | 0.598 |
| well | 43 (43.4) | 63 (56.8) |  |  | 46 (48.9) | 60 (51.7) |  |  | 56 (56.0) | 50 (45.5) |  |  | 47 (50.5) | 59 (50.4) |  |  | 40 (46.5) | 66 (53.2) |  |
| moderate | 36 (36.4) | 30 (27.0) |  |  | 29 (30.9) | 37 (31.9) |  |  | 27 (27.0) | 39 (35.5) |  |  | 24 (25.8) | 42 (35.9) |  |  | 30 (34.9) | 36 (29.0) |  |
| poor | 20 (20.2) | 18 (16.2) |  |  | 19 (20.2) | 19 (16.4) |  |  | 17 (17.0) | 21 (19.0) |  |  | 22 (23.7) | 16 (13.7) |  |  | 16 (18.6) | 22 (17.8) |  |
| pT stage |  |  | 0.308 |  |  |  | 0.860 |  |  |  | 0.951 |  |  |  | 1.000 |  |  |  | 0.578 |
| T1-2 | 89 (89.9) | 105 (94.6) |  |  | 86 (91.5) | 108 (93.1) |  |  | 93 (93.0) | 101 (91.8) |  |  | 86 (92.5) | 108 (92.3) |  |  | 81 (94.2) | 113 (91.1) |  |
| T3-4 | 10 (10.1) | 6 (5.4) |  |  | 8 (8.5) | 8 (6.9) |  |  | 7 (7.0) | 9 (8.2) |  |  | 7 (7.5) | 9 (7.7) |  |  | 5 (5.8) | 11 (8.9) |  |
| pN stage |  |  | 0.921 |  |  |  | 0.965 |  |  |  | 1.000 |  |  |  | 0.578 |  |  |  | 0.316 |
| 0 | 68 (68.7) | 78 (70.3) |  |  | 66 (70.2) | 80 (69.0) |  |  | 70 (70.0) | 76 (69.1) |  |  | 67 (72.0) | 79 (67.5) |  |  | 56 (65.1) | 90 (72.6) |  |
| 1-3 | 31 (31.3) | 33 (29.7) |  |  | 28 (29.8) | 36 (31.0) |  |  | 30 (30.0) | 34 (30.9) |  |  | 26 (28.0) | 38 (32.5) |  |  | 30 (34.9) | 34 (27.4) |  |
| Stage |  |  | 0.790 |  |  |  | 0.964 |  |  |  | 0.127 |  |  |  | 0.455 |  |  |  | 0.182 |
| I | 64 (64.6) | 74 (66.7) |  |  | 63 (67.0) | 75 (64.7) |  |  | 68 (68.0) | 70 (63.7) |  |  | 64 (68.8) | 74 (63.2) |  |  | 51 (59.3) | 87 (70.2) |  |
| II | 13 (13.1) | 16 (14.4) |  |  | 12 (12.8) | 17 (14.7) |  |  | 10 (10.0) | 19 (17.3) |  |  | 11 (11.8) | 18 (15.4) |  |  | 17 (19.8) | 12 (9.7) |  |
| III | 18 (18.2) | 19 (17.1) |  |  | 16 (17.0) | 21 (18.0) |  |  | 21 (21.0) | 16 (14.5) |  |  | 17 (18.3) | 20 (17.1) |  |  | 15 (17.4) | 22 (17.7) |  |
| IV | 4 (4.1) | 2 (1.8) |  |  | 3 (3.2) | 3 (2.6) |  |  | 1 (1.0) | 5 (4.5) |  |  | 1 (1.1) | 5 (4.3) |  |  | 3 (3.5) | 3 (2.4) |  |

NSCLC, non-small cell lung cancer; AD, adenocarcinoma; SqCC, squamous cell carcinoma; ADSq, adenosquamous carcinoma; VEGF, vascular endothelial growth factor
