# Supplementary material for: Tumor-associated macrophage, angiogenesis and lymphangiogenesis markers predict prognosis of non-small cell lung cancer patients
Source: J Transl Med. 2020 Nov 23;18:443. doi: 10.1186/s12967-020-02618-z (PMC7686699; doi:10.1186/s12967-020-02618-z)
Supplement: Supplementary file 2 — Additional file 2: Fig. S1 Association between tumor-associated macrophage and VEGFs in human non-small cell lung cancer (NSCLC). Correlation between tumor-associated macrophage and VEGF-A (a) and VEGF-C (b). Fig. S2 Correlation among tumor-associated macrophage-, angiogenesis- and lymphangiogenesis-related markers in patients with adenocarcinoma NSCLC. CD68 expression positively correlated with CD163 (a), VEGF-A (b), and VEGF-C (c) expression. CD163 expression showed a significant positive correlation with VEGF-A (d), but a weak correlation with VEGF-C (e) expression. There is a positive correlation between VEGF-A and VEGF-C (f). Fig. S3 Correlation among tumor-associated macrophage-, angiogenesis- and lymphangiogenesis-related markers in patients with squamous cell carcinoma NSCLC. CD68 expression positively correlated with CD163 (a), VEGF-A (b), and VEGF-C (c) expression. CD163 expression showed a moderate correlation with VEGF-A (d) and VEGF-C (e) expression. There is a significant positive correlation between VEGF-A and VEGF-C (f). Fig. S4 Kaplan–Meier survival curves for tumor-associated macrophage-, angiogenesis- and lymphangiogenesis-related markers in NSCLC patients with adenocarcinoma. Patients expressing CD68 had better overall survival than patients who did not (OS rate, 85.9% vs. 75.7%, log rank p = 0.034) (a), while CD163 was not associated with patient survival (p = 0.470) (b). Patients with a high M2 ratio (CD163/CD68) had significantly shorter overall survival than patients with a low M2 ratio (OS rate, 76.0% vs. 84.5%, log rank p = 0.047) (c). There were no meaningful overall survival differences for VEGF-A (p = 0.092) (d) and VEGF-C (p = 0.899) (e) expression in NSCLC patients. Fig. S5 Kaplan–Meier survival curves for tumor-associated macrophage-, angiogenesis- and lymphangiogenesis-related markers in NSCLC patients with squamous cell carcinoma. There were no meaningful overall survival differences for CD 68 (p = 0.153) (a), CD163 (p = 0.449) (b), [file 12967_2020_2618_MOESM2_ESM.pdf]

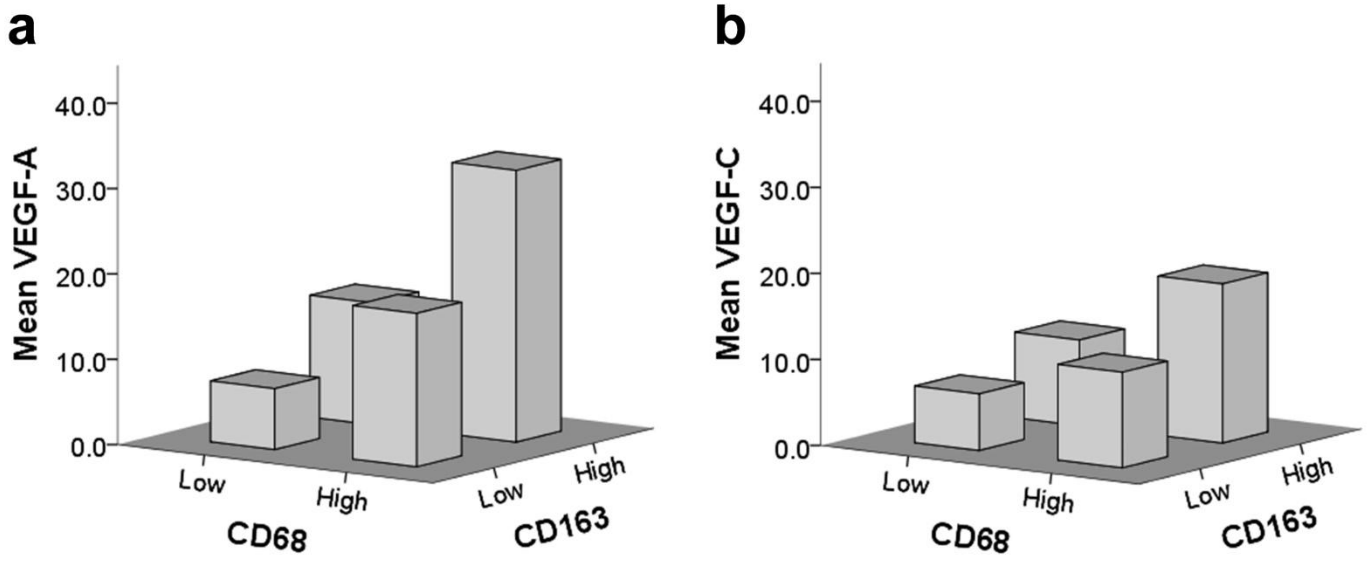

Supplementary Fig. S1 Association between tumor-associated macrophage and VEGFs in human non-small cell lung cancer (NSCLC). Correlation between tumor-associated macrophage and VEGF-A (**a**) and VEGF-C (**b**).

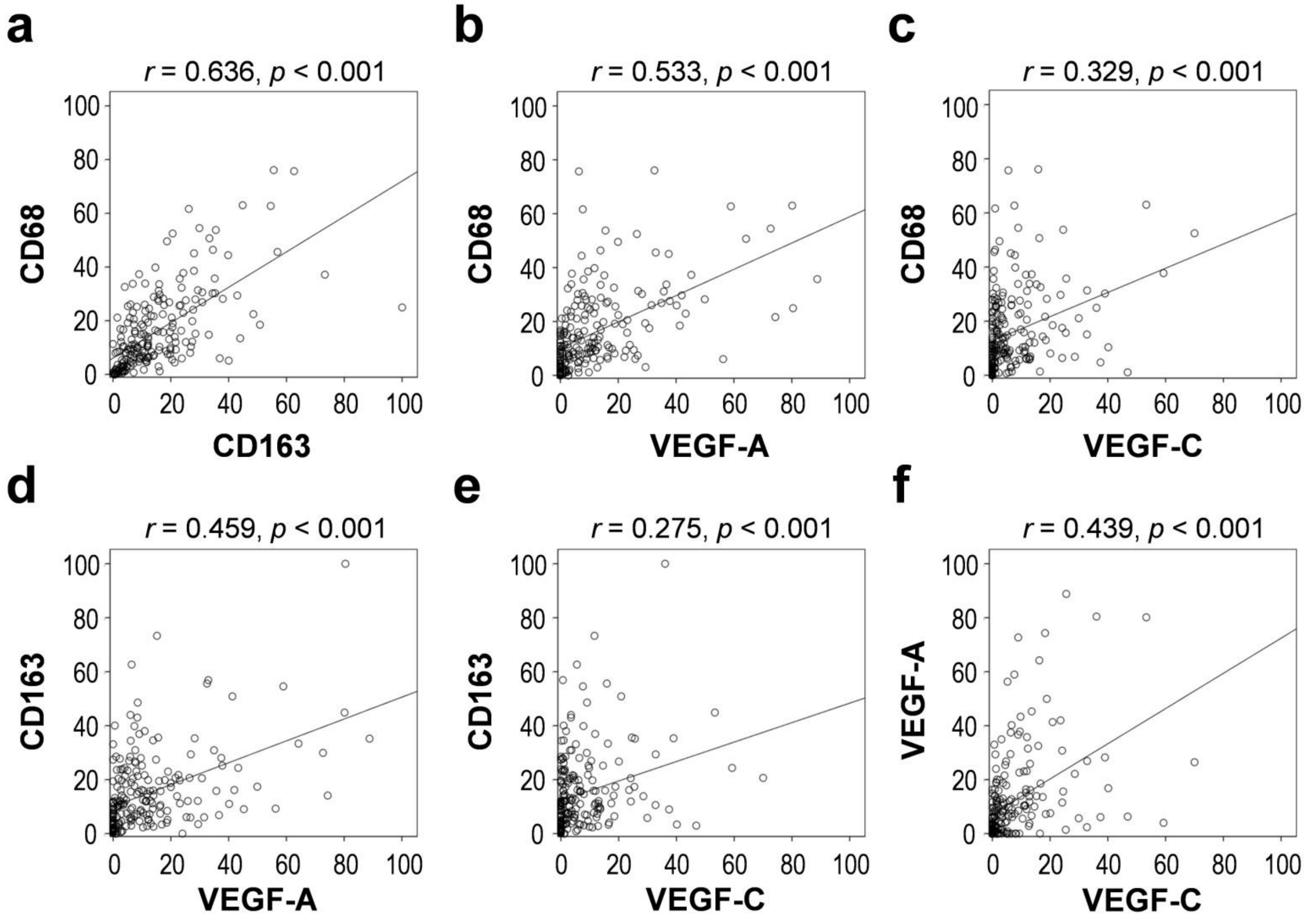

Supplementary Fig. S2 Correlation among tumor-associated macrophage-, angiogenesis- and lymphangiogenesis-related markers in patients with adenocarcinoma NSCLC. CD68 expression positively correlated with CD163 (**a**), VEGF-A (**b**), and VEGF-C (**c**) expression. CD163 expression showed a significant positive correlation with VEGF-A (**d**), but a weak correlation with VEGF-C (**e**) expression. There is a positive correlation between VEGF-A and VEGF-C (**f**).

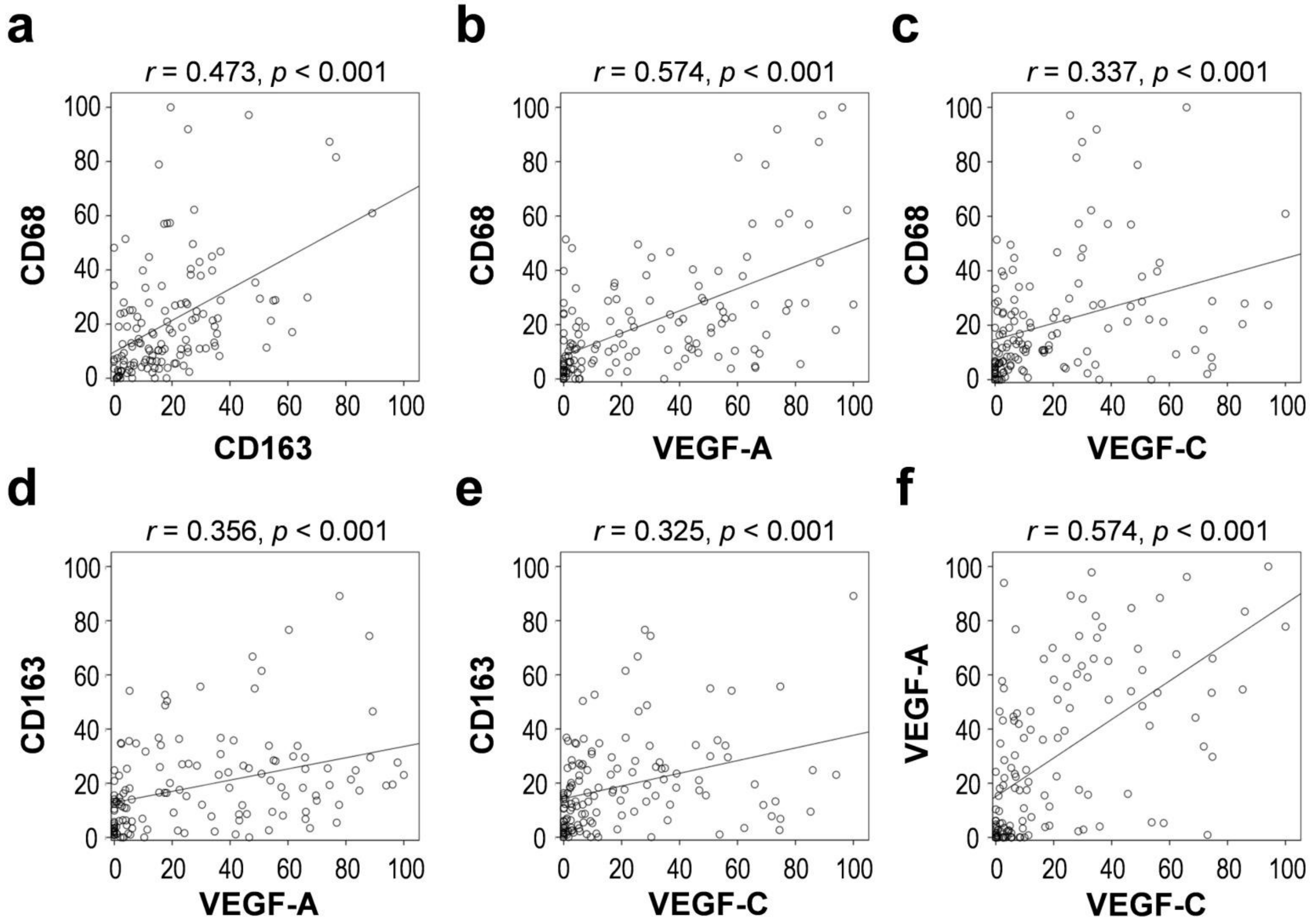

Supplementary Fig. S3 Correlation among tumor-associated macrophage-, angiogenesis- and lymphangiogenesis-related markers in patients with squamous cell carcinoma NSCLC. CD68 expression positively correlated with CD163 (**a**), VEGF-A (**b**), and VEGF-C (**c**) expression. CD163 expression showed a moderate correlation with VEGF-A (**d**) and VEGF-C (**e**) expression. There is a significant positive correlation between VEGF-A and VEGF-C (**f**).

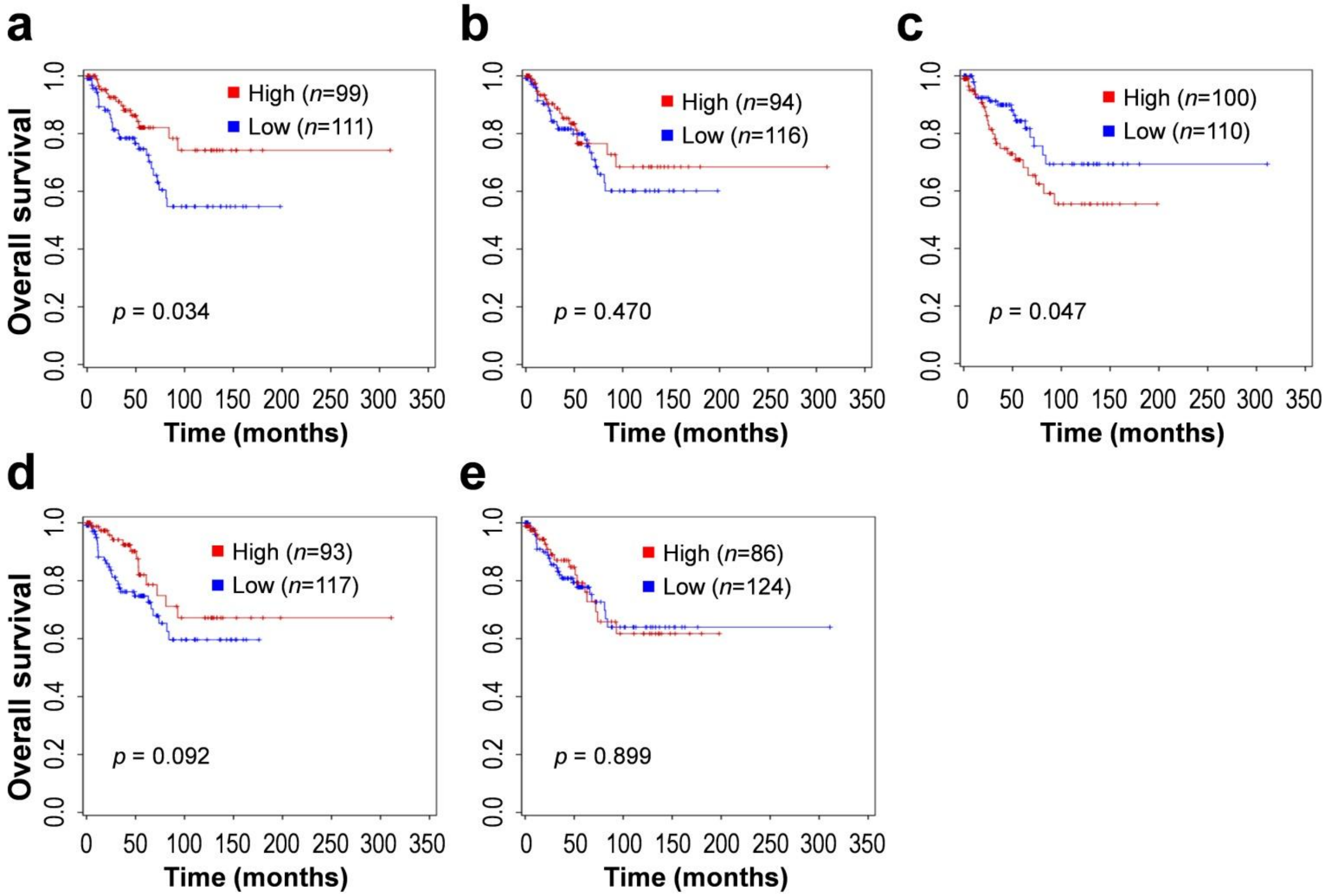

Supplementary Fig. S4 Kaplan-Meier survival curves for tumor-associated macrophage-, angiogenesis- and lymphangiogenesis-related markers in NSCLC patients with adenocarcinoma. Patients expressing CD68 had better overall survival than patients who did not (OS rate, 85.9% *vs.* 75.7%, log rank p=0.034) (**a**), while CD163 was not associated with patient survival (p=0.470) (**b**). Patients with a high M2 ratio (CD163/CD68) had significantly shorter overall survival than patients with a low M2 ratio (OS rate, 76.0% *vs.* 84.5%, log rank p=0.047) (**c**). There were no meaningful overall survival differences for VEGF-A (p=0.092) (**d**) and VEGF-C (p=0.899) (**e**) expression in NSCLC patients.

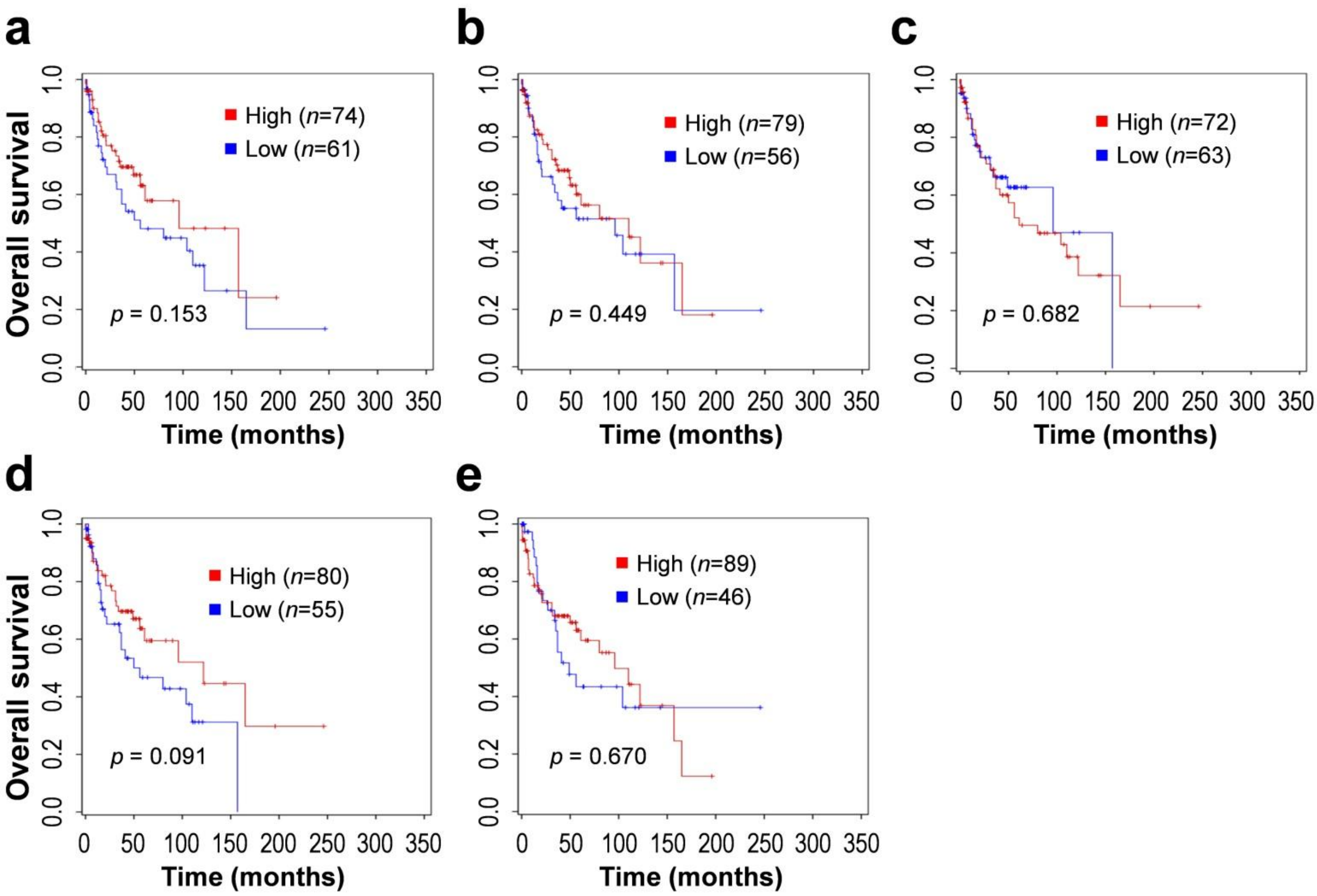

Supplementary Fig. S5 Kaplan-Meier survival curves for tumor-associated macrophage-, angiogenesis- and lymphangiogenesis-related markers in NSCLC patients with squamous cell carcinoma. There were no meaningful overall survival differences for CD 68 (p=0.153) (**a**), CD163 (p=0.449) (**b**), M2 ratio (p=0.682) (**c**), VEGF-A (p=0.091) (**d**) and VEGF-C (p=0.670) (**e**) expression in NSCLC patients with squamous cell carcinoma.

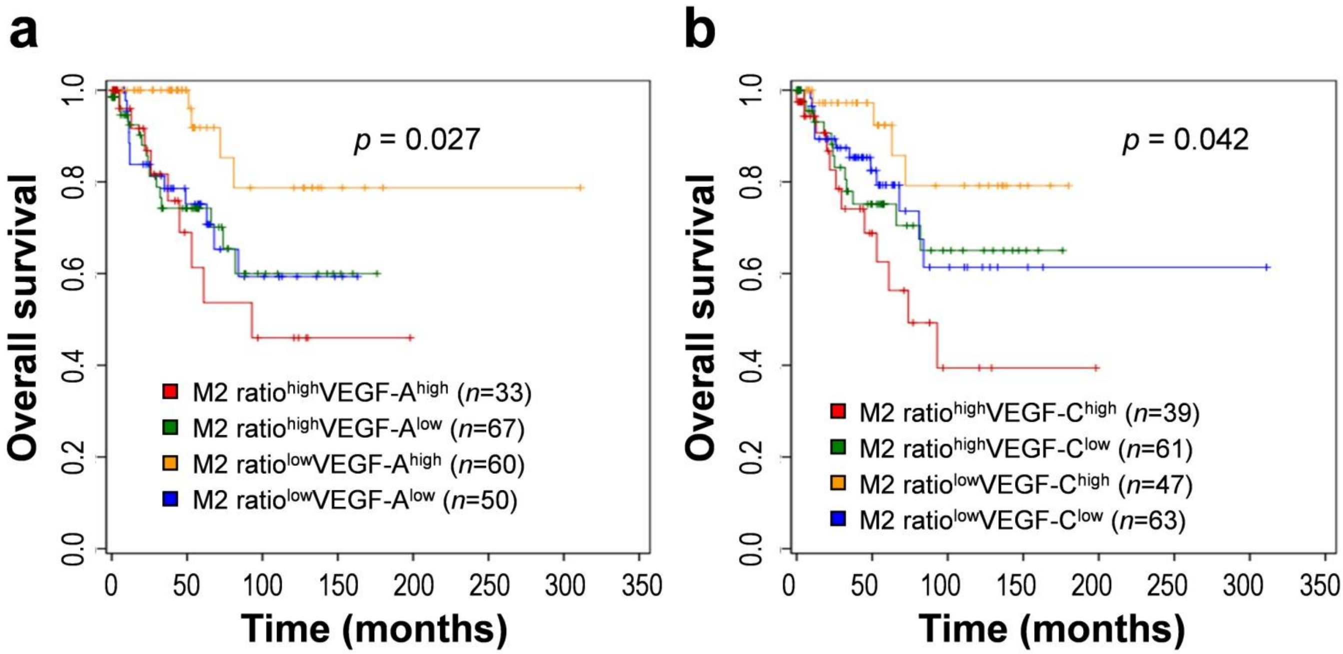

Supplementary Fig. S6 Survival analysis of NSCLC patients with M2 ratio expression according to angiogenesis (VEGF-A) or lymphangiogenesis (VEGF-C) marker expression in NSCLC patients with adenocarcinoma. Survival differences were observed among 4 NSCLC patient groups classified according to their M2 ratio and VEGF-A expression (log rank p=0.027) (**a**). A significant difference of survival rate was found among 4 NSCLC patient groups classified according to their M2 ratio and VEGF-C expression (log rank p=0.042) (**b**).

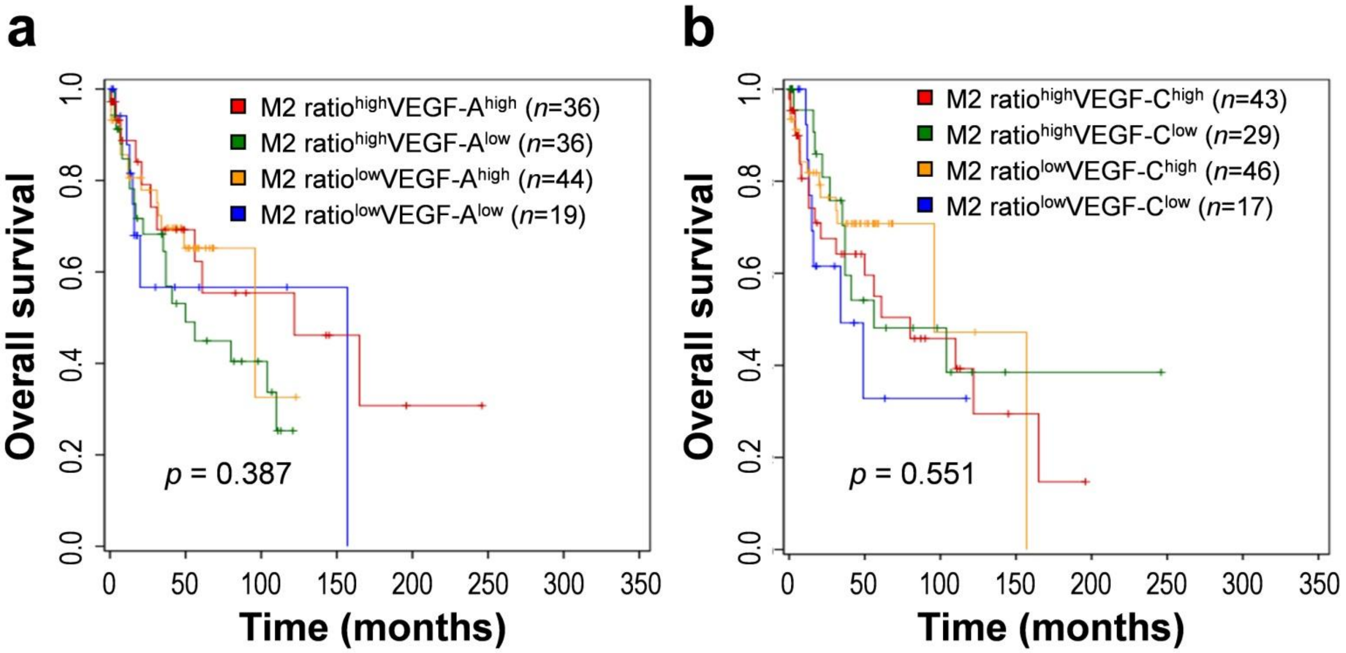

Supplementary Fig. S7 Survival analysis of NSCLC patients with M2 ratio expression according to angiogenesis (VEGF-A) or lymphangiogenesis (VEGF-C) marker expression in NSCLC patients with squamous cell carcinoma. There were no meaningful overall survival differences for combination M2 ratio and VEGF-A (p=0.387) (**a**) or VEGF-C (p=0.551) (**b**).
